# Time-efficient and beneficial strategy: low-volume high-intensity interval training for cardiometabolic health and body composition outcomes in children and adolescents with overweight or obesity—a systematic review and meta-analysis

**DOI:** 10.3389/fphys.2025.1732253

**Published:** 2026-01-30

**Authors:** Chenxin Ye, Honghao Fu, Xiao Zhou, Zhi Zhang

**Affiliations:** School of Physical Education, Huazhong University of Science and Technology, Wuhan, China

**Keywords:** Adolescent, body composition, cardiorespiratory fitness, low-volume high-intensity interval training, obesity, time-efficient exercise

## Abstract

**Objectives:**

This meta-analysis aimed to synthesize evidence on the effects of low-volume high-intensity interval training (LV-HIIT) on body composition and cardiometabolic health in overweight and obese children and adolescents. Specifically, we sought to: (1) quantify the effects of LV-HIIT *versus* non-exercise controls; (2) directly compare LV-HIIT with moderate-intensity continuous training (MICT); (3) identify participant- and program-level moderators prespecified subgroup and meta-regression analyses, to inform time-efficient pediatric exercise prescriptions.

**Methods:**

Six databases (PubMed, EMBASE, Cochrane Library, Web of Science, CNKI, and EBSCO) were systematically searched. Randomized controlled trials comparing LV-HIIT with either MICT or inactive controls in participants aged 7–16 years with overweight or obesity were included. Using random-effects models to calculate standardized mean differences with 95% confidence intervals Subgroup and meta-regression analyses were conducted to identify potential moderators.

**Results:**

Twelve trials involving 609 participants were included. Compared with non-exercise controls, LV-HIIT reduced BMI (SMD = −1.15, 95% CI [−1.68, −0.61]), body fat percentage (SMD = −0.84, 95% CI [−1.09, −0.59]), waist circumference (SMD = −0.62, 95% CI [−0.93, −0.32]), systolic blood pressure (SMD = −0.80, 95% CI [−1.11, −0.49]), diastolic blood pressure (SMD = −0.47, 95% CI [−0.77, −0.17]), while increasing VO_2_max (SMD = 2.10, 95% CI [1.32, 2.87]). Compared with MICT, LV-HIIT showed greater improvements in BMI (SMD = −0.27, 95% CI [−0.49, −0.04]), systolic blood pressure (SMD = −0.25, 95% CI [−0.55, −0.05]), and VO_2_max (SMD = 0.76, 95% CI [0.39, 1.13]), while showing comparable effectiveness in reducing body fat percentage (SMD = −0.06, 95% CI [−0.35, 0.22]) and waist circumference (SMD = −0.37, 95% CI [−0.75, 0.01]). Subgroup analyses revealed greater BMI reductions with LV-HIIT among participants who were overweight at baseline and among males. Meta-regression with baseline adiposity as a continuous moderator indicated participants with lower baseline BMI may experience greater BMI reductions after LV-HIIT.

**Conclusion:**

LV-HIIT significantly improves body composition, cardiometabolic health, and cardiorespiratory fitness in overweight and obese children and adolescents, offering comparable or superior benefits to traditional MICT in approximately half the time.

**Systematic Review Registration:**

https://www.crd.york.ac.uk/PROSPERO/view/CRD420250655540.

## 1 Introduction

Overweight and obesity among children and adolescents have become significant global public health challenges. Currently, more than 390 million youths are classified as overweight or obese, and the prevalence continues to rise at an alarming rate. These trends are consistent with public health analyses that have highlighted obesity as a major contributor to the non-communicable disease burden and emphasized the need for comprehensive, population-level prevention strategies (Gill, 1997; Kopelman and Albon, 1997). Obesity is closely associated with cardiovascular and metabolic health risks (Zhang et al., 2024). Obese adolescents frequently have high blood pressure, abnormal blood glucose, and dyslipidemia. These factors increase their long-term risk of cardiovascular diseases in adulthood (Juonala et al., 2011; McPhee et al., 2020). Studies have shown that compared to their peers with healthy weights, overweight or obese children and adolescents are more likely to remain obese in adulthood. This not only increases the prevalence of obesity-related diseases but also raises the overall adult obesity rate (Llewellyn et al., 2016; Simmonds et al., 2015; Simmonds et al., 2016).

Early intervention in obesity is essential to reduce future disease risk. Physical activity (PA) is a key method for prevention and management owing to its positive effect on cardiovascular and metabolic health (Dongsheng and Haipeng, 2011). Recent guidelines suggest that children and adolescents aged 5–17 years should engage in an average of 60 min of moderate to vigorous PA daily to maintain and improve metabolic health (Bull et al., 2020). However, international data indicate that over 80% of children and adolescents fail to meet the prescribed PA criteria (Rhodes et al., 2020). Moderate-intensity continuous training (MICT) has traditionally been used to increase PA, showing significant effects on reducing body fat and the risk of metabolic syndrome in obese children (Alberga et al., 2013; O'Donoghue et al., 2021; Thorogood et al., 2011). However, MICT requires long periods of exercise. Modern teenagers face academic pressure and increased recreational activities (Stiglic and Viner, 2019). Given that lack of time and access to facilities are commonly reported barriers to participation in PA (Biddle et al., 2011; Gray et al., 2016), it is necessary to explore more efficient exercise strategies suitable for obese children and adolescents.

High-intensity interval training (HIIT) is an efficient exercise method gaining increasing popularity among fitness enthusiasts, competitive athletes, and in the field of public health (Buchheit and Laursen, 2013; Milanović et al., 2015; Poon et al., 2021). It consists of short bursts of high-intensity activity alternating with rest periods or low-intensity exercise (Coates et al., 2023). Many studies show that HIIT can bring significant physiological improvements in a short time, including enhancing cardiorespiratory endurance, reducing body fat, and improving cardiovascular metabolic health (Gist et al., 2014; Martin-Smith et al., 2020; Stensvold et al., 2020). In obese adults, HIIT has been proven to achieve fat loss and cardiorespiratory fitness (CRF) improvement effects that are no less than or even better than MICT (Maillard et al., 2018; Deng and Wang, 2024). Additionally, its more efficient training mode can reduce the required weekly exercise time by approximately 40% (Wewege et al., 2017).

Among overweight and obese adolescents, both MICT and HIIT can improve body composition and cardiovascular metabolic health. However, their relative effectiveness remains debated. Studies have demonstrated that HIIT can reduce the cardiovascular metabolic risk factors of obese children and adolescents (da Silva et al., 2020; Racil et al., 2016), and even has a better effect on body fat percentage (Miguet et al., 2020). In contrast, Morrissey et al. indicate that its efficacy is not significantly different from MICT (Morrissey et al., 2018). Cvetković et al. found that MICT achieves greater improvement in obese children and adolescents (Cvetkovic et al., 2018). Regarding body composition and metabolic health, several studies show that HIIT is more effective than MICT (Julian et al., 2022; Miguet et al., 2020). However, a meta-analysis found that MICT is more effective than HIIT in improving body composition indicators (Liu et al., 2020). Notably, an 8-week trial in sedentary obese adolescents showed equivalent improvements in adiposity with both HIIT and MICT (Sun et al., 2024). This underscores the persistent lack of consensus regarding the relative superiority of these methods. Nevertheless, high-intensity interval training has shown promise in reducing cardiometabolic risk in obese adolescents, though most protocols require substantial time commitment (Racil et al., 2023).

Traditional HIIT programs have long exercise durations, which limit their applicability to young people with limited time. A meta-analysis of 67 HIIT intervention studies showed that longer training sessions or higher weekly training durations increase participants’ dropout rates (Reljic et al., 2019). Recent guidelines and cohort studies have emphasized the benefits of short-duration, low-volume exercise for inducing physiological adaptations (Ho et al., 2018; Little et al., 2019; Padulo et al., 2016; Yin M. et al., 2025; Yin M. Y. et al., 2024). Specifically, sprint interval training (SIT) and other time-efficient protocols have been shown to effectively improve cardiorespiratory fitness and body composition in adolescents with overweight or obesity, offering a feasible alternative to continuous exercise (Delgado-Floody et al., 2019; González-Gálvez et al., 2024; Salus et al., 2022). This approach helps promote participation in physical activities by reducing the duration of each exercise session. Therefore, research on the health benefits of such short-duration exercises has become a highly focused and popular field of study. To make HIIT better fit public health needs, experts suggest shifting to more time-saving training programs that still provide sufficient load stimulation (Vollaard and Metcalfe, 2017; Yin M. et al., 2025). Evidence suggests that even at lower volume and shorter duration, LV-HIIT achieves comparable improvements in CRF, blood pressure, and other health outcomes relative to traditional aerobic training or higher-volume HIIT, even surpassing them (Lu et al., 2025; Sabag et al., 2022; Vollaard et al., 2017; Weston M. et al., 2014). This efficient approach is expected to improve exercise compliance while providing similar cardiovascular metabolic improvement effects. Therefore, exploring LV-HIIT is crucial in optimizing obesity intervention measures for children and adolescents.

Currently, there is no consensus on a precise definition of LV-HIIT, and inconsistencies in classification methods impede the direct comparison of results across studies. For this study, we align with Taylor et al. and Sabag et al. by defining LV-HIIT as the total duration spent in active intervals (excluding rest periods) of less than 15 min (Taylor et al., 2019). This approach provides a consistent metric that accommodates individual differences while allowing researchers to tailor the warm-up, cool-down, and recovery phases to specific study designs. By adopting this definition, we aim to streamline comparisons and reduce perceived heterogeneity.

To date, several systematic reviews have explored the effects of LV-HIIT on adult health (Sultana et al., 2019; Yin M. et al., 2024). However, there is no systematic review or meta-analysis on the benefits of this interval training programs for obese adolescents and children regarding body composition and cardiovascular metabolic health. Thus, by systematically evaluating existing data through meta-analysis, this study aims to determine whether LV-HIIT is a feasible and effective intervention for improving body composition and enhancing cardiopulmonary health among overweight or obese adolescents, providing scientific evidence to guide future exercise prescriptions and inform public health policies.

## 2 Methods

### 2.1 Protocol and registration

This review was conducted following established systematic review methodology for exercise interventions and reported according to the Preferred Reporting Items for Systematic Reviews and Meta-Analyses (PRISMA) 2020 statement (Impellizzeri and Bizzini, 2012; Page et al., 2021). The protocol was prospectively registered in the PROSPERO database (ID: CRD420250655540).

### 2.2 Search strategy

Searches were conducted in PubMed (MEDLINE), EMBASE, the Cochrane Library, Web of Science, CNKI, and EBSCO. The search was last updated on 11 August 2025, and no date restrictions were applied during database searching. Medical Subject Headings (MeSH) terms utilized included “high-intensity interval training,” “body composition,” “adolescents,” and “children,” along with their respective synonyms. Specifically, these MeSH terms were combined with free-text keywords related to high-intensity interval training (e.g., “high-intensity interval training,” “high-intensity intermittent exercise,” “sprint interval training,” “low-volume high-intensity interval training,” “LV-HIIT,” “HIIT,” “HIIE”) and body-composition or cardiometabolic indices (e.g., “BMI”, “waist circumference”, “hip circumference”, “waist-to-hip ratio”, “body-fat percentage”, “lean body mass”, “blood pressure”, “maximal oxygen uptake”, “physical fitness”, “CRF”, “peak VO_2_”, “metabolic-syndrome Z-score”). Inclusion was limited to English-language randomized controlled trials (RCTs) that provided pre- and post-intervention data on body composition or cardiorespiratory variables. We manually searched the reference lists of relevant papers and previous reviews to identify any additional eligible studies. All retrieved records were screened against predetermined inclusion and exclusion criteria.

### 2.3 Inclusion and exclusion criteria

Eligibility criteria were predefined based on the PICOS framework (Population, Intervention, Comparison, Outcomes, and Study design). To maximize the inclusion of all potentially relevant studies, no time restriction was imposed during the literature search. Subsequent rigorous screening based on predefined eligibility criteria ensured the relevance and methodological quality of the included studies, thereby strengthening the comprehensiveness and validity of the findings. (1) population: According to the WHO 2007 Growth Reference for children and adolescents aged 5–19 years (de Onis et al., 2007), this review included children (5–12 years) and adolescents (13–19 years) with no professional training background (i.e., no structured training for ≥3 months before enrollment) and free of musculoskeletal disorders or other clinical contraindications to exercise (Cole et al., 2000); (2) intervention: LV-HIIT using aerobic modes (e.g., walking, running, cycling), prescribed at a minimum of vigorous intensity (≥77% of maximum heart rate (HRmax) or rating of perceived exertion (RPE) ≥14 on the 6–20 scale) and accumulating <15 min of total time in active high-intensity intervals per session (recovery not counted) (Garber et al., 2011; Sultana et al., 2019; Taylor et al., 2019; Weston K. S. et al., 2014). This threshold was selected to standardize the duration of the true physiological stimulus while ensuring the protocols remain feasible within time-constrained settings, such as school physical education classes, with warm-up and cool-down permitted; MICT was defined as exercising at 64%–76% HRmax, or 46%–63% of maximal oxygen uptake (VO_2_max), or 40%–59% of heart rate reserve (HRR), or with RPE of 12–13 (Williams et al., 2019). (3) comparison: a no-exercise control or a MICT comparator using aerobic modalities; (4) outcomes: availability of pre- and post-intervention quantitative data for at least one prespecified endpoint related to body composition (e.g., body mass index, fat mass, body-fat percentage, waist circumference, hip circumference, waist-to-hip ratio) or cardiorespiratory health (e.g., systolic blood pressure, diastolic blood pressure, VO_2_ max, CRF, metabolic-syndrome Z-score); and (5) study design: parallel-group RCTs (including randomized crossover trials with pre–post comparisons), conducted in humans, published as full-length articles in English, and implementing supervised exercise interventions (defined as sessions conducted under the direct observation of researchers, physical education teachers, or qualified instructors to ensure protocol fidelity) lasting at least 4 weeks with a minimum frequency of two sessions per week.

Studies were excluded if they: combined LV-HIIT with other training modalities or dietary interventions such that the isolated effect of LV-HIIT could not be determined; used non-aerobic primary modalities; were non-randomized, single-arm, acute, or unsupervised interventions; enrolled adults or normal-weight youths when data were inseparable; lacked sufficient statistics to calculate effect sizes; or were non-English, conference abstracts, protocols, reviews, animal, or *in vitro* studies.

### 2.4 Study selection

According to the PICOS criteria, one researcher (CY) independently used EndNote 21 software to remove duplicate records. The deduplicated literature was then exported and provided to two independent researchers (HF and XZ), who screened the titles and abstracts based on the predefined inclusion and exclusion criteria. In case of disagreement, a third independent researcher (ZZ) reviewed the articles to determine their inclusion status. Any discrepancies were subsequently resolved through discussion in accordance with the established protocol. Additionally, potential sources of relevant articles included references from previous systematic reviews on the topic and the professional knowledge of the research team. These sources helped identify articles that might meet the inclusion criteria of this review but were not initially captured in the literature search.

### 2.5 Data extraction

Two reviewers (HF and CY) independently extracted information from each eligible study, recording details such as the first author, publication year, participant profile, study design, and training regimen. We also collected data on adherence, dropout rates, and any reported adverse events. A third reviewer (XZ) verified the extracted data to ensure its accuracy and completeness. All discrepancies between reviewers were resolved through discussion to achieve consensus. The extracted details included: (1) publication information (first author, year, country, and study setting); (2) participant characteristics (sample size, sex, age, and weight status); (3) intervention characteristics (type of exercise, program duration, training frequency, session duration, and other relevant details); and (4) outcome data (mean ± SD) for both the experimental group (EG) and control group (CG) before and after the intervention. If data were missing or presented only graphically, the authors were contacted to request the necessary information. Corresponding authors of three studies were contacted to request missing information; two of them responded and provided usable additional data. Studies for which missing data could not be obtained were excluded from the final analysis.

### 2.6 Assessment of study quality and risk of bias

Two reviewers (HF and CY) independently assessed the risk of bias. Any disagreements were resolved through discussion, and if consensus could not be reached, a third reviewer (XZ) acted as an arbitrator. The risk of bias was assessed using the Cochrane Collaboration’s Risk of Bias Tool 2 (RoB2) (Sterne et al., 2016), which evaluates random sequence generation, random allocation concealment, blinding of outcome assessment, incomplete outcome data, and selective outcome reporting. Additionally, the Physiotherapy Evidence Database (PEDro) scale was used to assess the risk of bias and methodological quality of the included studies (de Morton, 2009). The PEDro scale scores studies on a scale of 0–10, with scores ≥6 considered high quality, 4–5 considered moderate quality, and ≤3 considered low quality.

### 2.7 Statistical analysis

#### 2.7.1 Data synthesis and effect measures

We extracted the means, standard deviations (SD), and sample sizes for each group before and after the intervention. Effects were summarised using the pre-to-post-intervention difference (mean ± SD) for each outcome measure. First, the mean differences from pre-to-post-intervention were calculated for each intervention group using the following formula:

$${MD}_{diff}=M_{post}-M_{pre}$$

Where $M_{diff}$ represents the raw mean difference, $M_{post}$ is the reported mean post-intervention, and $M_{pre}$ is the reported mean pre-intervention.

The SD of the change scores was calculated according to the formula provided by the Cochrane Handbook for Systematic Reviews of Interventions (version 6.3) (Cumpston et al., 2019a):

$${SD}_{change}=\sqrt{{SD}_{baseline}^{2}+{SD}_{final}^{2}-\left(2\times Corr\times {SD}_{final}\right)}$$

${SD}_{change}$ denotes the standard deviation of change scores, ${SD}_{\;baseline}$ and ${SD}_{final}$ represent the standard deviations at pre- and post-intervention, respectively, and Corr denotes the correlation coefficient between pre- and post-intervention measurements.

We then pooled the MD and SD in a combined analysis. Pairwise meta-analyses were conducted for each outcome to compare the relative efficacy of various exercise interventions. The effect size was quantified as the standardized mean difference (SMD) with its 95% confidence interval (CI). All statistical analyses were performed using a random-effects model in Stata 17.0 (StataCorp, College Station, TX, USA). To ensure high-quality graphical representation, data visualization was conducted using R software (Nakagawa et al., 2023; Viechtbauer, 2010), based strictly on the effect estimates and confidence intervals calculated in Stata. Effect sizes were classified according to the Cochrane Handbook: values of 0.20–0.49 indicated a small effect, 0.50–0.79 a moderate effect, and >0.80 a significant effect. Moreover, *r* is the pre-post test correlation coefficient. Correlation coefficients for pre- and post-intervention were rarely reported in the included studies and were generally assumed to be *r* = 0.50, as suggested by the Cochrane Handbook (Cumpston et al., 2019b). Although we analysed the existing data, we did not receive responses from the study authors regarding the reported data. Therefore, we adopted the conservative value of 0.5 for the calculations. Sensitivity analyses were performed using correlation coefficients of 0.30 and 0.70 to assess the robustness of effect estimates to this assumption (Supplementary Table S3) (Impellizzeri and Bizzini, 2012). A *p*-value <0.05 was considered statistically significant.

Statistical heterogeneity among studies was assessed using the *I* ^*2*^ statistic, Cochran’s Q test, and the between-study variance (τ^2^). *I* ^*2*^ was interpreted as 0%–40% might not be important, 30%–60% may represent moderate heterogeneity, 50%–90% substantial heterogeneity, and 75%–100% considerable heterogeneity. These thresholds are not absolute and should be interpreted in light of the magnitude and direction of effects, as well as the overall strength of evidence for heterogeneity. For random-effects models, τ^2^ was estimated and reported alongside *I* ^*2*^ to quantify the absolute extent of between-study variance. A *p*-value ≤0.10 for Cochran’s Q test was considered indicative of statistically significant heterogeneity. Sensitivity analyses were performed to evaluate the robustness of the results (Cumpston et al., 2019b). Funnel plots were used to visually assess publication bias. Each study was deleted from the model once to analyse its influence on the overall results. Egger regression tests were performed to detect minor study effects and possible publication bias (Higgins and Thompson, 2002). Subgroup analyses were conducted when pronounced heterogeneity was detected to explore its potential causes. Descriptive analyses were performed where appropriate.

#### 2.7.2 Subgroup and meta-regression analysis

This study employed subgroup and meta-regression analyses to explore sources of heterogeneity among studies and potential moderating factors, and conducted statistical analyses of the variables. The following variables were included in the subgroup analysis: (1) baseline BMI, (2) age, (3) gender, and (4) Intervention period. For baseline BMI, a pragmatic cut-off of 30 kg·m^−2^ was used to distinguish trials with comparatively lower *versus* higher obesity severity within the overweight/obese range (i.e., BMI <30 kg·m^−2^ vs. BMI ≥30 kg·m^−2^). This threshold was selected because absolute BMI was reported in all studies, whereas BMI z-scores were inconsistently reported, and therefore absolute BMI served as the only common metric across trials. It is important to note that these categorizations are exploratory and do not replace age- and sex-specific paediatric criteria for overweight and obesity.

To explore the dose–response effects of LV-HIIT on BMI and body fat percentage, we conducted a set of meta-regression analyses based on random effects models, each including the modified variables associated with the protocol: (1) repetitions, (2) duration per repetitions, (3) total session duration, (4) total duration per week, (5) baseline BMI and (6) high-intensity duration per session. Meta-regression analyses excluded studies with incomplete reporting of moderator variables. Available case analysis was used, with sample sizes indicated for each analysis. In addition, we used the contour-enhanced funnel plot combined with Egger’s asymmetry test to assess publication bias (Egger et al., 1997; Peters et al., 2008), with *p* > 0.05 indicating no significant publication bias. For all other statistical analyses, a *p*-value <0.05 was considered statistically significant.

### 2.8 Certainty of evidence

The risk of bias was considered in the interpretation of the results by applying the Grading of Recommendations Assessment, Development, and Evaluation (GRADE) methodology, which rates the certainty of evidence as “high” (further research is improbable to change our confidence in the estimate of effect), “moderate” (further research is likely to have an important impact on our confidence in the estimate of effect and may change the estimate), “low” (further research is very likely to have an important impact on our confidence in the estimate of effect and is likely to change the estimate) or “very low” (any estimate of effect is very uncertain) (Schünemann et al., 2019). GRADE assessments were completed by one reviewer (CY) and reviewed by a second reviewer (ZZ).

## 3 Results

### 3.1 Search results

**FIGURE 1:**
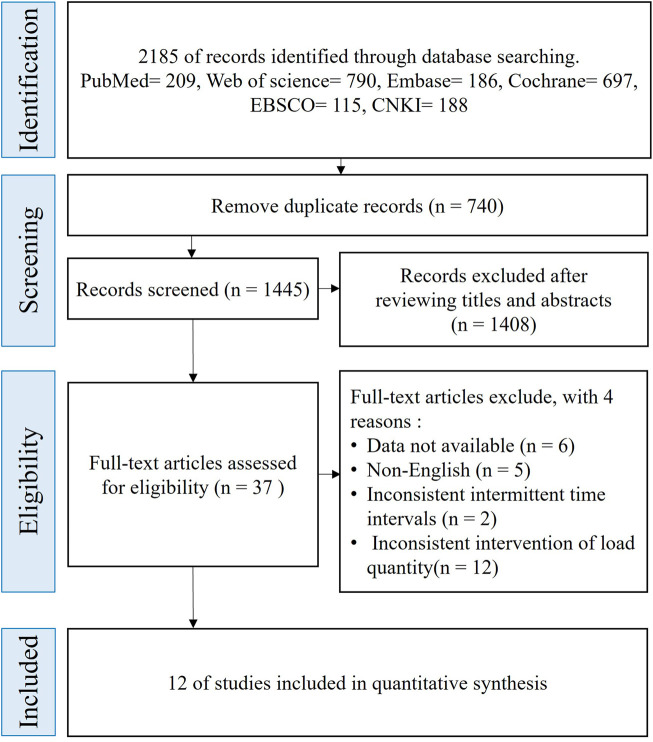
PRISMA flow diagram for included and excluded studies.

The flow diagram illustrating the trial selection process is depicted in Figure 1. The initial literature search yielded 2,185 potentially eligible articles; after removing 740 duplicates, 1,445 records remained for further screening. Following title and abstract screening, 1,408 articles were excluded, resulting in 37 studies selected for full-text assessment. Ultimately, 12 trials fulfilled the predefined eligibility criteria and were included in the final meta-analysis.

### 3.2 Characteristics of included studies

Participant characteristics across the included studies are summarized in Table 1. A comprehensive description of the intervention protocols is presented in Table 2. The studies were published between 2018 and 2025, with seven conducted in Asia, four in Africa, and one in Australia and Europe. The included trials involved a total of 609 overweight or obese participants, whose mean ages ranged from 7 to 16 years. Four studies exclusively recruited girls, four exclusively recruited boys, two included both genders, and the two study did not report the gender distribution of participants (Cao et al., 2022a; Li et al., 2023). Baseline demographic data included BMI (reported in all 12 trials) and body fat percentage (reported in 11 trials; not reported in Su et al., 2024). Ten studies compared the effectiveness of LV-HIIT and MICT, two compared LV-HIIT with non-exercising control groups, and five studies incorporated both MICT and no-exercise control conditions. Intervention durations ranged from 8 to 12 weeks, with most lasting 12 weeks (n = 8). Exercise sessions were performed two to three times weekly. Exercise modalities consisted primarily of running-based or football-based drills. In LV-HIIT and MICT protocols, training intensity was prescribed based on HRmax, HRpeak, HRR, VO_2_max, VO_2_peak, or Maximum Aerobic Speed (MAS). LV-HIIT sessions involved 4–12 min of high-intensity exercise, preceded by a 5–18-min warm-up at 80%–100% HRmax and followed by a 5–15-min cool-down. Participants in the MICT groups exercised for 12–40 min per session, with each session preceded by a 5–15-min warm-up at 55%–80% HRmax and concluded with a 5–15-min cool-down. Regarding outcome assessments, BMI was derived from height and weight measurements; height was measured using calibrated stadiometers across all included studies, while weight was assessed using calibrated electronic scales in two studies, with the remaining studies utilizing diagnostic scales or body composition analyzers. Body fat percentage was evaluated by bioelectrical impedance analysis (BIA) in six studies, dual-energy X-ray absorptiometry (DXA) in four studies, and skinfold thickness in one study. Cardiorespiratory fitness (VO_2_max) was determined using direct gas analysis systems in four studies, while three studies utilized indirect field-based prediction equations (e.g., 20-m shuttle run test) (Leger et al., 1988). Resting blood pressure was monitored using automatic electronic devices in six studies and manual sphygmomanometers in two studies. Detailed device specifications, measurement protocols, and reliability data for each study are provided in Supplementary Table S4.

**TABLE 1:** Participant characteristics.

Study	Groups	Subjects	B/G	Age (years)	BMI (kg/m^2^)	Body fat (%)
Abassi et al. (2025)	LV-HIIT	17	0/17	16.7 ± 1.3	33.1 ± 4.1	34.5 ± 3.8
	CON	16	0/16	17.3 ± 1.1	33.6 ± 5.4	32.7 ± 2.7
Su et al. (2024)	LV-HIIT	22	22/0	15.0 ± 1.0	32.1 ± 1.2	NR
	MICT	22	22/0	14.0 ± 1.0	31.1 ± 1.2	NR
Cao et al. (2024)	LV-HIIT	14	7/7	12.4 ± 0.4	24.4 ± 1.0	37.9 ± 2.7
	MICT	14	7/7	12.1 ± 0.6	23.8 ± 1.3	38.1 ± 1.1
	CON	14	7/7	12.4 ± 0.6	23.8 ± 1.3	37.2 ± 2.4
Zuo et al. (2023)	LV-HIIT	20	20/0	8.1 ± 0.9	21.6 ± 1.8	21.8 ± 5.9
	MICT	20	20/0	7.9 ± 0.6	21.6 ± 1.4	19.3 ± 5.5
Abassi et al. (2023)	LV-HIIT	13	0/13	6.4 ± 1.2	32.6 ± 3.6	33.7 ± 3.4
	MICT	13	0/13		33.1 ± 5.6	33.8 ± 2.9
	CON	12	0/12		33.2 ± 5.7	33.0 ± 3.0
Li et al. (2023)	LV-HIIT	16	NR	11.0 ± 0.8	24.2 ± 1.3	35.4 ± 5.8
	MICT	16	NR		24.3 ± 1.1	36.5 ± 5.1
	CON	16	NR		23.6 ± 0.6	35.8 ± 4.9
Abassi et al. (2022)	LV-HIIT	13	13/0	16.7 ± 0.2	32.6 ± 3.6	33.7 ± 3.3
	MICT	13	13/0		33.1 ± 5.6	33.8 ± 2.8
	CON	12	12/0		33.2 ± 5.7	33.0 ± 3.0
Cao et al. (2022a)	LV-HIIT	17	NR	10.8 ± 0.7	24.3 ± 2.2	38.4 ± 4.0
	MICT	16	NR		24.4 ± 0.9	39.2 ± 2.7
	CON	18	NR		23.8 ± 0.8	38.3 ± 1.6
Cao et al. (2022b)	LV-HIIT	12	12/0	11.4 ± 0.8	24.5 ± 1.1	38.4 ± 4.0
	MICT	11	11/0	11.2 ± 0.7	24.4 ± 0.9	39.2 ± 2.7
	CON	13	13/0	11.0 ± 0.7	23.8 ± 0.8	38.3 ± 1.6
Cao et al. (2022c)	LV-HIIT	20	10/10	11.2 ± 0.7	23.4 ± 1.6	44.2 ± 8.1
	CON	20	10/10	10.9 ± 0.4	23.8 ± 1.5	43.6 ± 7.2
Paahoo et al. (2020)	LV-HIIT	15	15/0	11.1 ± 0.9	25.3 ± 1.0	28.0 ± 1.4
	MICT	15	15/0	10.8 ± 1.0	25.0 ± 0.6	27.8 ± 1.0
	CON	15	15/0	11.2 ± 0.9	25.0 ± 1.8	27.8 ± 1.0
Cvetkovic et al. (2018)	LV-HIIT	10	10/0	11–13	26.6 ± 3.3	32.8 ± 8.2
	MICT	11	11/0		25.4 ± 4.0	36.2 ± 6.7
	CON	14	14/0		25.2 ± 4.7	29.9 ± 8.4

Abbreviations: BMI, body mass index; CON, non-exercising control, B boy, G girl, LV-HIIT, low-volume high-intensity interval training; MICT, moderate-intensity continuous training; NR, not reported; SD, standard deviation; SEM, standard error of the mean.

Values were reported as *mean ± SD*; in instances where results were presented as *mean ± SEM*, *SEM* was converted to *SD* using *SD* = *SEM* × square root of populations ( Sqrt∧n ).

**TABLE 2:** Intervention protocol.

Study	Groups	Mode	Warm-up	Duration	Intensity	Cool-down	Total high intensity/session	Frequency	Duration (weeks)	Total training time
Abassi et al. (2025)	LV-HIIT	Running	15 min	6–8 × 30 s	90%–95% HRmax	15 min	8 min	2	9	38 min
	CON	NR	NR	NR	NR	NR	NR	NR	NR	NR
Su et al. (2024)	LV-HIIT	Running	5 min	9 × 1 min	85%–95% HRmax	5 min	9 min	3	8	19 min
	MICT	Running	5 min	35 min	65%–75% HRmax	5 min	35 min	3	8	45 min
Cao et al. (2024)	LV-HIIT	Running	5 min	3× (8 × 15 s)	90% HRmax	5 min	12 min	3	12	22 min
	MICT	Running	5 min	3× (8 × 15 s)	70% HRmax	5 min	12 min	3	12	22 min
Zuo et al. (2023)	LV-HIIT	Running	5 min	2× (15 × 20 s)	80%–85% HRmax	5 min	10 min	3	8	20 min
	MICT	Running	5 min	30 min	55%–60% HRmax	5 min	30 min	3	8	40 min
Abassi et al. (2023)	LV-HIIT	Running	15 min	2× (6–8 × 30 s)	100–110% MAS	15 min	6–8 min	3	12	36–38 min
	MICT	Running	15 min	2× (6–8 × 30 s)	70–80% MAS	15 min	6–8 min	3	12	36–38 min
	CON	NR	NR	NR	NR	NR	NR	NR	NR	NR
Li et al. (2023)	LV-HIIT	Running	NR	2× (8 × 15 s)	80%–100% HRmax	NR	4 min	3	12	NR
	MICT	Running	NR	20–40 min	70%–80% HRmax	NR	20–40 min	3	12	NR
	CON	NR	NR	NR	NR	NR	NR	NR	NR	NR
Abassi et al. (2022)	LV-HIIT	Running	15 min	2× (6–8 × 30 s)	100–110% MAS	10 min	6–8 min	3	12	31–33 min
	MICT	Running	15 min	2× (6–8 × 30 s)	70–80% MAS	10 min	6–8 min	3	12	31–33 min
	CON	NR	NR	NR	NR	NR	NR	NR	NR	NR
Cao et al. (2022a)	LV-HIIT	Running	NR	3× (8 × 15 s)	80%–100% HRmax	NR	12 min	3	12	NR
	MICT	Running	NR	20–40 min	70%-80% HRmax	NR	20–40 min	3	12	NR
	CON	NR	NR	NR	NR	NR	NR	NR	NR	NR
Cao et al. (2022b)	LV-HIIT	Running	5 min	2× (8 × 15 s)	80%–81% HRmax	5 min	4 min	3	12	14 min
	MICT	Running	5 min	30 min	70%–71% HRmax	5 min	30 min	3	12	40 min
	CON	NR	NR	NR	NR	NR	NR	NR	NR	NR
Cao et al. (2022c)	LV-HIIT	Running	15 min	3× (8 × 15 s)	80%–90% HRmax	5 min	12 min	3	12	32 min
	CON	NR	NR	NR	NR	NR	NR	NR	NR	NR
Paahoo et al. (2020)	LV-HIIT	Running	18 min	3× (10 × 10 s)	100%–110% MAS	8 min	5 min	3	12	31 min
	MICT	Running	15 min	30 min	40%–70% HRR	5 min	30 min	3	12	50 min
	CON	NR	NR	NR	NR	NR	NR	NR	NR	NR
Cvetkovic et al., 2018	LV-HIIT	Running	10 min	3× (5–10) × (15 s–20 s)	100% MAS	10 min	4 min30 s-10 min	3	12	24–30 min
	MICT	Football	10 min	4 × 8 min	80% HRmax	10 min	32 min	3	12	52 min
	CON	NR	NR	NR	NR	NR	NR	NR	NR	NR

Abbreviations: CON, non-exercising control; LV-HIIT, low-volume high-intensity interval training; MICT, moderate-intensity continuous training; NR, not reported; SD, standard deviation; SEM, standard error of the mean; HRmax, Maximum heart rate; MAS maximum aerobic speed; HRR, heart rate reserve.

Total training time calculated as warm-up + high-intensity intervals + rest intervals + cool-down. *NR*, indicates insufficient information was provided to calculate total session duration.

### 3.3 Methodological quality of included studies

The PEDro scores ranged from moderate to high quality (5–9) for the systematic review and meta-analysis. Table 3 provides a detailed summary of the methodological quality assessment, including individual PEDro scores for each study. However, none of the included trials reported test–retest reliability statistics (e.g., intraclass correlation coefficients) for the primary cardiometabolic outcomes such as BMI, body fat, blood pressure, or cardiorespiratory fitness. Most studies described measurement procedures in detail and, in some cases, cited external validation studies for the devices used, but within-study reliability estimates were not provided.

**TABLE 3:** Methodological quality of included studies assessed using the physiotherapy evidence database (PEDro) scale.

Study	Eligibility criteria	Random allocation	Concealed allocation	Similar baseline	Participant blinding	Investigator blinding	Assessor blinding	Completeness of follow-up	Intention to treat	Between-group comparisons	Point measures and variability	Total score
Abassi et al. (2025)	Yes	0	0	1	0	0	0	1	1	1	1	5
Su et al. (2024)	Yes	1	1	1	0	1	1	1	1	1	1	9
Cao et al. (2024)	Yes	1	1	1	0	0	0	1	1	1	1	7
Zuo et al. (2023)	Yes	1	0	1	0	0	1	0	1	1	1	6
Abassi et al. (2023)	Yes	0	0	1	0	0	0	1	1	1	1	5
Li et al. (2023)	Yes	1	1	1	0	0	0	0	1	1	1	6
Abassi et al. (2022)	Yes	0	0	1	0	0	0	1	1	1	1	5
Cao et al. (2022a)	Yes	1	0	1	0	0	0	1	1	1	1	6
Cao et al. (2022b)	Yes	1	0	1	0	0	1	1	1	1	1	7
Cao et al. (2022c)	Yes	1	0	1	0	0	1	1	1	1	1	7
Paahoo et al. (2020)	Yes	0	0	1	0	0	0	1	1	1	1	6
Cvetkovic et al. (2018)	Yes	0	0	1	0	0	0	1	1	1	1	6

Studies scoring ≥6 is considered high quality, those scoring 4–5 are considered moderate quality, and those scoring ≤3 are considered low quality.

1. Eligibility criteria were specified (not included in the total score).

2. Subjects were randomly allocated to groups (in a crossover study, subjects were randomly allocated an order in which treatments were received).

3. Allocation was concealed.

4. The groups were similar at baseline regarding the most important prognostic indicators.

5. There was blinding of all subjects.

6. There was blinding of all therapists who administered the therapy.

7. There was blinding of all assessors who measured at least one key outcome.

8. Measures of at least one key outcome were obtained from more than 85% of the subjects initially allocated to groups.

9. All subjects for whom outcome measures were available received the treatment or control condition as allocated or, where this was not the case, data for at least one key outcome was analyzed by “intention to treat”.

10. The results of between-group statistical comparisons are reported for at least one key outcome.

11. The study provides both point measures and measures of variability for at least one key outcome.

### 3.4 Effects of LV-HIIT on health outcomes compared with no training

Ten trials including 298 participants assessed the effects of LV-HIIT *versus* non-exercising control on body composition, CRF and blood pressure (Abassi et al., 2023; Abassi et al., 2025; Abassi et al., 2022; Cao et al., 2022a; 2022b; 2022c; Cao et al., 2024; Cvetkovic et al., 2018; Li et al., 2023; Paahoo et al., 2020). These studies assessed outcomes including body composition, CRF, and cardiometabolic markers. Regarding feasibility, exercise adherence was reported in four studies for the LV-HIIT groups (91%–100%) (Cao et al., 2022a; 2022b; Li et al., 2023; Paahoo et al., 2020). Retention was also high across the included trials, with dropout rates ranging from 0% to 28%. Notably, the majority of studies (8 out of 10) reported a dropout rate of 20% or lower, and no exercise-related adverse events were reported, collectively supporting the feasibility of LV-HIIT for adolescents with obesity.

#### 3.4.1 Body composition

**FIGURE 2:**
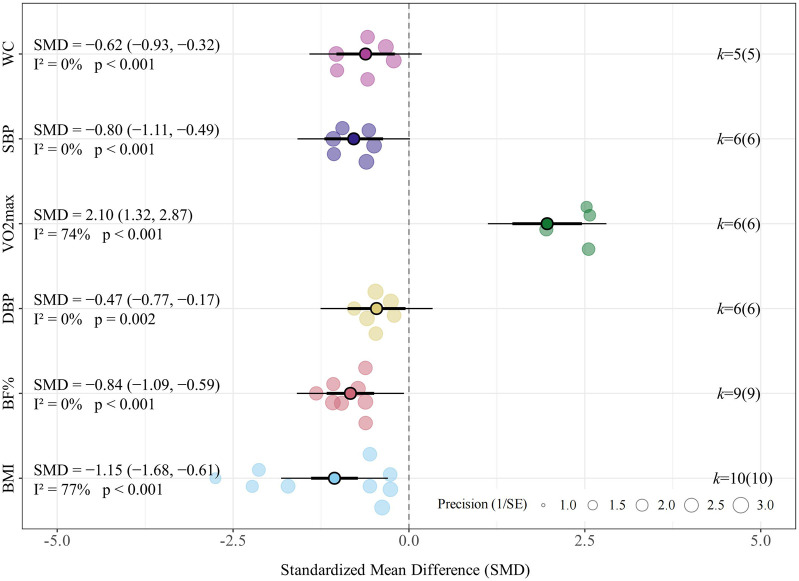
Summary of the impact of LV-HIIT vs. CON on health outcomes.

LV-HIIT consistently yielded beneficial effects across all evaluated body composition outcomes. Specifically, the meta-analysis found a significant improvement effect of LV-HIIT compared with the control group on BMI (SMD = −1.15, 95% CI [−1.68, −0.61], *p* < 0.001, *I* ^*2*^ = 77.4%, *τ* ^*2*^ = 0.597; Figure 2), corresponding to an estimated absolute reduction of approximately 2.0 kg/m^2^. Furthermore, significant reductions were observed for body fat percentage (SMD = −0.84, 95% CI [−1.09, −0.59], *p* < 0.001, *I* ^*2*^ = 0.0%, *τ* ^*2*^ < 0.001; Figure 2) and waist circumference (SMD = −0.62, 95% CI [−0.93, −0.32], *p* < 0.001, *I* ^*2*^ = 0.0%, *τ* ^*2*^ < 0.001; Figure 2).

Subgroup analysis showed that LV-HIIT produced greater BMI reductions in the prespecified strata (Table 4). Gender significantly moderated the effect (between-subgroup *p* = 0.01): the pooled SMD was larger in males (*Hedges’ g* = −1.07)than in females (*Hedges’ g* = −0.50). Baseline BMI further acted as a significant moderator (*p* = 0.03), with greater BMI reductions at lower baseline levels (≤30 kg·m^−2^, *Hedges’ g* = −1.45) compared to higher levels (>30 kg·m^−2^, *Hedges’ g* = −0.50). Across age groups, LV-HIIT was associated with lower BMI in both children aged ≤12 years (*Hedges’ g* = −1.31) and individuals aged >12 years (*Hedges’ g* = −0.91). However, age did not significantly moderate the overall effect (between-subgroup *p* = 0.47). Regarding body fat percentage, LV-HIIT yielded significant reductions compared to the control group across all reported subgroups (all pooled *p* < 0.01; Table 5), with no statistically significant differences between subgroups (between-subgroup *p* > 0.05 for all). Given the limited number of studies within several strata, these subgroup findings should be considered exploratory hypothesis-generating analyses and interpreted with caution.

**TABLE 4:** The effect of LV-HIIT *versus* CON on BMI: subgroup and moderation meta-analyses.

Subgroup	K	Hedges’ *g*	95% CI	*p*_d_	*I* ^2^	*Cochran’s Q*	*p*_b_
Age							
≤12	6	−1.31	[−2.08, −0.55]	<0.01	81.6%	<0.01	0.47
>12	4	−0.91	[−1.69, −0.13]	0.02	71.3%	<0.01	
Gender							
Male	3	−1.07	[−2.48, 0.35]	0.14	0	<0.01	0.01
Female	3	−0.50	[−0.94, −0.06]	0.03	0	0.92	
Mixed	4	−1.73	[−2.41, −1.04]	<0.01	0	0.03	
Baseline BMI							
≤30	7	−1.45	[−2.17, −0.73]	<0.01	81.3%	<0.01	0.03
>30	3	−0.50	[−0.94, −0.06]	0.03	0	0.92	

K, the total number of effects included in the pooled effect size; Hedges’ *g*, the effect size indicators used in the pooled; 95%CI, 95% confidence interval; *I* ^2^, quantitative indicators of heterogeneity; *p*_d_, overall pooled effect; *p*_b_, between-subgroup difference.

**TABLE 5:** The effect of LV-HIIT *versus* CON on body fat percentage: subgroup and moderation meta-analyses.

Subgroup	K	Hedges’ *g*	95% CI	*p*_d_	*I* ^2^	*Cochran’s Q*	*p*_b_
Age							
≤12	5	−0.85	[−1.18, −0.53]	<0.01	0%	0.74	0.93
>12	4	−0.83	[−1.22, −0.44]	<0.01	0%	0.56	
Gender							
Male	2	−1.04	[−1.61, −0.48]	<0.01	0	0.83	0.60
Female	3	−0.68	[−1.12, −0.24]	<0.01	0	0.98	
Mixed	4	−0.87	[−1.23, −0.52]	<0.01	0	0.40	
Baseline BMI							
≤30	6	−0.92	[−1.22, −0.62]	<0.01	0%	0.66	0.38
>30	3	−0.68	[−1.12, −0.24]	<0.01	0%	0.97	

K, the total number of effects included in the pooled effect size; Hedges’ *g*, the effect size indicators used in the pooled; 95%CI, 95% confidence interval; *I* ^2^, quantitative indicators of heterogeneity; *p*_d_, overall pooled effect; *p*_b_, between-subgroup difference.

Meta-regression analyses were conducted to explore the modifying effects of repetition number, duration per repetition, total duration per week, baseline BMI, and high-intensity duration per session on the effectiveness of LV-HIIT in reducing BMI and body fat percentage. However, we did not find a significant association between any participant characteristics and training variables and the effects of LV-HIIT on BMI and body fat percentage (*p* > 0.05 for all; Supplementary Figure S1). Notably, when treating baseline BMI as a continuous moderator, we observed a borderline trend at the threshold of significance (*p* = 0.05; Supplementary Figure S1), indicating that lower baseline BMI is associated with greater reductions in BMI in response to LV-HIIT. Importantly, this borderline association is based on only ten data points and may represent a spurious finding. This hypothesis requires confirmation in future adequately powered analyses with individual participant data.

#### 3.4.2 Cardiovascular health and CRF

Regarding systolic blood pressure (SBP), the meta-analysis indicated a significant reduction following LV-HIIT compared to no-exercise controls (SMD = −0.80, 95% CI [−1.11, −0.49], *p* < 0.001, *I* ^*2*^ = 0.0%, τ^2^ < 0.001; Figure 2), corresponding to an absolute reduction of approximately 3.5 mmHg. For diastolic blood pressure (DBP), LV-HIIT produced a moderate reduction compared with controls (SMD = −0.47, 95% CI [−0.77, −0.17], *p* = 0.002, *I* ^*2*^ = 0.0%, *τ* ^2^ < 0.001; Figure 2), equivalent to a decrease of approximately 1.8 mmHg. Relative to no-exercise controls, participants in the LV-HIIT group demonstrated significant increases in VO_2_max (SMD = 2.10, 95% CI [1.32, 2.87], *p* < 0.001, *I* ^*2*^ = 73.6%, τ^2^ = 0.566; Figure 2).

### 3.5 Effects of LV-HIIT on health outcomes compared with MICT

Ten trials involving a total of 302 participants were included in the direct comparison of LV-HIIT *versus* MICT (Abassi et al., 2023; Abassi et al., 2022; Cao et al., 2022a; 2022b; Cao et al., 2024; Cvetkovic et al., 2018; Li et al., 2023; Paahoo et al., 2020; Su et al., 2024; Zuo et al., 2023). These studies assessed outcomes including body composition, CRF, and cardiometabolic markers. Regarding feasibility, retention was high across the included trials, with dropout rates ranging from 0% to 29%. Notably, the vast majority of studies (9 out of 10) reported a dropout rate of 20% or lower, and no exercise-related adverse events were reported in either group. Furthermore, exercise adherence was explicitly reported in six studies (Cao, Tang, et al., 2022a; 2022b; Li et al., 2023; Paahoo et al., 2020; Su et al., 2024; Zuo et al., 2023), with comparable high rates observed for both LV-HIIT (91%–100%) and MICT (84%–97%). These findings suggest that LV-HIIT is as feasible and tolerable as traditional moderate-intensity training for adolescents with obesity.

#### 3.5.1 Body composition

**FIGURE 3:**
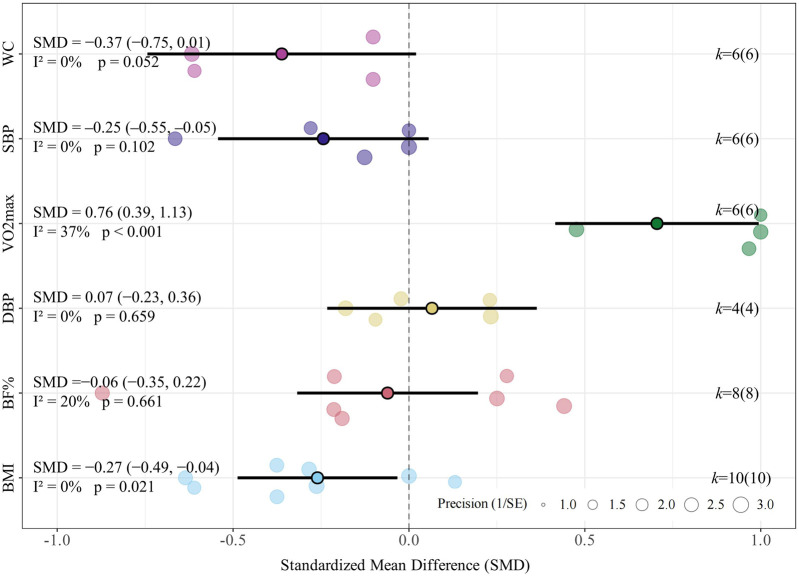
Summary of the impact of LV-HIIT vs. MICT on health outcomes.

Compared with MICT, LV-HIIT demonstrated a slight reduction in BMI (SMD = −0.27, 95% CI [−0.49, −0.04], *p* = 0.021, *I* ^*2*^ = 0.0%, τ^2^ < 0.001; Figure 3). However, no significant differences were found between LV-HIIT and MICT regarding body fat percentage (SMD = −0.06, 95% CI [−0.35, 0.22], *p* = 0.661, *I* ^*2*^ = 20.1%, τ^2^ = 0.034; Figure 3) and waist circumference (SMD = −0.37, 95% CI [−0.75, 0.01], *p* = 0.052; *I* ^*2*^ = 0.0%, τ^2^ < 0.001; Figure 3). The clinical significance of the small BMI difference (SMD = −0.24) is uncertain, as the 95% CI barely excluded zero and the effect would be classified as small by conventional thresholds.

No statistically significant differences were observed in BMI and body fat percentage among all subgroups (all between-subgroup *p* > 0.05; Tables 6 and 7). A small but statistically significant pooled effect favoring LV-HIIT for reducing BMI was found in interventions lasting more than 8 weeks (*Hedges’ g* = −0.27), whereas shorter programs showed no significant difference.

**TABLE 6:** The effect of LV-HIIT *versus* MICT on BMI: subgroup and moderation meta-analyses.

Subgroup	K	Hedges’ *g*	95% CI	*p*_d_	*I* ^2^	Cochran’s *Q*	*p*_b_
Age							
≤12	6	−0.18	[−0.47, 0.12]	0.24	0%	0.95	0.50
>12	4	−0.33	[−0.69, 0.02]	0.07	0%	0.70	
Gender							
Male	4	−0.15	[−0.49, 0.19]	0.40	0%	0.86	0.75
Female	3	−0.35	[−0.81, 0.11]	0.13	0%	0.97	
Mixed	3	−0.29	[−0.70, 0.12]	0.17	0%	0.46	
Baseline BMI							
≤30	7	−0.24	[−0.51, 0.04]	0.09	0%	0.88	0.97
>30	3	−0.25	[−0.65, 0.16]	0.23	0%	0.75	
Intervention period							
≤8	2	−0.17	[−0.59, 0.26]	0.44	0%	0.68	0.68
>8	8	−0.27	[−0.54, 0.00]	0.04	0%	0.92	

K, the total number of effects included in the pooled effect size; Hedges’ *g*, the effect size indicators used in the pooled; 95%CI, 95% confidence interval; *I* ^2^, quantitative indicators of heterogeneity; *p*_d_, overall pooled effect; *p*_b_, between-subgroup difference.

**TABLE 7:** The effect of LV-HIIT *versus* MICT on body fat percentage: subgroup and moderation meta-analyses.

Subgroup	K	Hedges’ *g*	95%CI	*p*_d_	*I* ^2^	*Cochran’s Q*	*p*_b_
Age							
≤12	5	0.01	[−0.44, 0.47]	0.95	50.5%	0.08	0.48
>12	3	−0.21	[−0.65, 0.23]	0.34	0%	0.99	
Gender							
Male	3	−0.22	[−0.88, 0.43]	0.50	58.6%	0.08	0.39
Female	2	−0.22	[−0.77, 0.33]	0.42	0%	0.99	
Mixed	3	0.19	[−0.22, 0.60]	0.37	0%	0.44	
Baseline BMI							
≤30	6	−0.02	[−0.40, 0.36]	0.91	40.1%	0.14	0.56
>30	2	−0.22	[−0.77, 0.33]	0.42	0%	0.99	
Intervention period							
≤8	1	−0.06	[−0.67, 0.56]	0.86	0%	<0.01	0.97
>8	7	−0.07	[−0.41, 0.27]	0.69	31%	0.18	

K, the total number of effects included in the pooled effect size; Hedges’ *g*, the effect size indicators used in the pooled; 95%CI, 95% confidence interval; *I* ^2^, quantitative indicators of heterogeneity; *p*_d_, overall pooled effect; *p*_b_, between-subgroup difference.

#### 3.5.2 Cardiovascular health and CRF

Regarding SBP, the meta-analysis found no statistically significant difference between LV-HIIT and MICT (SMD = −0.25, 95% CI [–0.55, −0.05], *p* = 0.102, *I* ^*2*^ = 0.0%, τ^2^ < 0.001; Figure 3). For DBP, the meta-analysis found no significant differences in DBP (SMD = 0.07, 95% CI [−0.23, 0.36], *p* = 0.659, *I* ^*2*^ = 0.0%, τ^2^ < 0.001; Figure 3), indicating comparable effectiveness between LV-HIIT and MICT in DBP improvement. Compared to MICT, LV-HIIT also produced significant improvements in VO_2_max (SMD = 0.76, 95% CI [0.39, 1.13], *p* < 0.001, *I* ^*2*^ = 37.0%, τ^2^ < 0.001; Figure 3).

### 3.6 Assessment of publication bias

The risk of bias for each study is depicted in Supplementary Figure S2. Overall, the evidence quality for selective reporting and incomplete outcome data was relatively strong: approximately 70%–80% of the studies were assessed as having low risk of bias, indicating that the reporting of primary outcomes and the maintenance of data integrity were generally adequate. Allocation concealment was less consistently implemented, with roughly half or fewer rated as low risk, and the remainder mostly rated as unclear risk. Regarding blinding, participant/investigator and outcome assessor blinding were rated low risk in approximately half of the studies. At the same time, the rest were categorized as unclear risk, suggesting that caution is warranted when interpreting subjective measures or outcomes susceptible to expectation effects. For random sequence generation, most studies were rated as low risk. However, a notable proportion were designated as unclear due to insufficient detail regarding the specific methods or tools used to generate the random sequences and how they were implemented. Other sources of bias were predominantly rated as low risk. At the study level, the majority of trials demonstrated low or unclear risk of bias across all domains; only a few studies (e.g., Li et al., 2023) were assessed as having a high risk of bias in the “incomplete outcome data” domain, likely due to inadequate handling of dropouts or withdrawals, or deviations from the intended protocol.

The risk of publication bias was investigated using funnel plots combined with Egger’s test for the effects of included studies on body composition and cardiovascular health (Supplementary Figure S3). When ten or more studies were available, funnel plots combined with Egger’s regression test were used to quantitatively assess publication bias. In general, funnel plots showed symmetrical distributions of effect-size points, and Egger’s *p*-values predominantly exceeded 0.05, indicating no significant publication bias in analyses involving waist circumference, body fat percentage, diastolic blood pressure, or overall LV-HIIT vs. MICT comparisons. It should be noted that the funnel plots for BMI and VO_2_max comparisons against the control group showed an asymmetric aggregation pattern. This was corroborated by significant Begg’s and Egger’s test results, suggesting that small-study effects may have inflated these effect sizes. Due to the insufficient number of available studies, a reliable evaluation of publication bias was not feasible for fat mass or systolic blood pressure outcomes. In summary, systematic publication bias was not detected for most outcomes; however, the observed effects on BMI and VO_2_max should be interpreted cautiously and require validation through future pre-registered trials with larger sample sizes.

This study integrated the RoB 2 and PEDro tool evaluations to provide a more comprehensive assessment of the methodological quality and reliability of the included studies. The RoB 2 tool focuses on bias control and internal validity, while the PEDro tool emphasizes the structural quality of trial reports. Therefore, it is not uncommon for the scoring results to differ when using these two tools for assessment.

To comprehensively assess the certainty of evidence for each primary outcome indicator, we used the GRADE approach to evaluate the overall quality of the evidence (Supplementary Table S4). In the comparison between LV-HIIT and no training, outcomes such as body fat percentage, blood pressure, and WC were rated as moderate certainty, indicating a reasonable confidence level in the estimated effects. However, specific outcomes, including BMI and VO_2_max, were downgraded to low certainty due to inconsistent findings across studies or small sample sizes, leading to wide confidence intervals. In the comparison between LV-HIIT and MICT, VO_2_max was assigned a moderate certainty rating, while four outcomes, including BMI and blood pressure, were rated as low certainty primarily due to imprecision. Notably, WC was rated as very low certainty, owing to both limited sample sizes and serious imprecision. These GRADE assessments clarify the robustness of the current evidence and underscore the need for more rigorous, high-quality research in this area.

### 3.7 Sensitivity analysis

To assess the robustness of the study results, we applied a two-level random-effects model and the leave-one-out method to evaluate the influence of individual studies on the overall effect estimates. Each study was sequentially removed in the sensitivity analysis, and the pooled effect size was recalculated to determine whether the findings were materially affected. We further examined the impact of studies judged to have a high risk of bias or small sample sizes, based on PEDro scores, RoB 2 assessments, and the characteristics of the included studies. Excluding most of these studies did not substantially alter the combined effect size or its confidence interval. Notably, in the comparisons between LV-HIIT and the no-exercise control group and MICT, the leave-one-out analysis revealed that no single study significantly influenced the overall effect estimates.

In additional sensitivity analyses, we varied the assumed pre–post correlation coefficient used to impute change-score SDs (*r* = 0.30, 0.50, 0.70) and re-ran all primary random-effects meta-analyses (Supplementary Table S3). For most outcomes, the magnitude and direction of the pooled SMDs, as well as their statistical significance, were largely unchanged across different r values, indicating that the main findings were robust to this assumption. For waist circumference and SBP in the LV-HIIT *versus* MICT comparison, the point estimates consistently favoured LV-HIIT, but the 95% confidence intervals moved marginally across the line of no effect under different *r* assumptions, suggesting that these small between-group differences are statistically borderline and should be interpreted with caution. Overall, the convergence of the leave-one-out and correlation-based sensitivity analyses supports the robustness of the primary conclusions of this meta-analysis.

## 4 Discussion

To our knowledge, this systematic review and meta-analysis is the first to examine the effects of LV-HIIT on body composition and CRF specifically among obese adolescents. We aimed to compare the effectiveness of LV-HIIT interventions with MICT and non-exercise control conditions. Our key findings are: (1) LV-HIIT significantly improves body composition in obese adolescents. Compared to non-exercise controls, LV-HIIT participants experienced significant reductions in BMI, body fat percentage, and waist circumference. Compared to MICT, LV-HIIT showed only marginally greater reductions in BMI and waist circumference. However, differences in body fat percentage were minimal, indicating that LV-HIIT is equally effective as traditional aerobic training for fat control. (2) Regarding cardiovascular health indicators, LV-HIIT effectively lowered both systolic and diastolic blood pressure compared to the non-exercise control group. Compared to MICT, LV-HIIT demonstrated a modest advantage in reducing systolic blood pressure, though improvements in diastolic blood pressure were comparable between the two interventions. (3) The most pronounced benefit was observed in CRF. Participants in the LV-HIIT group achieved significant increases in VO_2_max compared to the non-exercise control group. Moreover, LV-HIIT also demonstrated substantial advantages over MICT in improving VO_2_max. (4) Our subgroup analyses indicated that the BMI-lowering effect of LV-HIIT was significantly moderated by sex and baseline BMI. In contrast, age did not significantly influence the outcome, suggesting comparable benefits for both younger (≤12 years) and older participants. Specifically, we observed that male participants achieved greater BMI reductions than females, and those with lower baseline BMI (overweight rather than obese) showed larger improvements, indicating that LV-HIIT may be especially effective for overweight youth and valuable in early obesity intervention. Consistent with these findings, our meta-regression analyses did not identify any participant characteristic or training parameter that significantly influenced the effectiveness of LV-HIIT. However, a borderline trend suggested that lower baseline BMI might predict a larger BMI reduction. These moderator findings should be interpreted with caution, as study-level meta-regression is susceptible to ecological fallacy and confounding by other unmeasured study characteristics, and such associations ideally need to be confirmed using adequately powered individual participant data meta-analyses. Collectively, these findings highlight LV-HIIT as an effective and time-efficient exercise modality for obese adolescents, offering significant practical implications for obesity management and cardiovascular disease prevention.

### 4.1 LV-HIIT is capable of enhancing cardiovascular health and CRF

HIIT is a time-efficient training strategy that rapidly improves physiological function and exercise performance, achieving enhancements comparable to those of traditional endurance training (Gibala et al., 2006). An increasing body of high-quality evidence indicates that HIIT significantly improves cardiorespiratory health in youth populations, especially among obese adolescents (Cao et al., 2019; Ortega et al., 2011). These improvements are equivalent to traditional aerobic training but require less time investment, offering greater efficiency (Corte de Araujo et al., 2012). From a physiological perspective, the brief vigorous bouts used in LV-HIIT fall within the high-intensity domain defined in current exercise physiology terminology, providing a substantial cardiometabolic load despite their short duration (Chamari and Padulo, 2015). Similar cardiorespiratory benefits have been consistently reported among adult populations (Wang et al., 2024). These observations align closely with the results of our meta-analysis. Specifically, our analysis revealed substantial improvements in VO_2_max among obese adolescents following LV-HIIT interventions. Martin-Smith et al. reported moderate improvements in adolescent CRF following HIIT (Men et al., 2023). Similarly, Martin-Smith et al. documented significant increases in VO_2_max following HIIT compared to inactivity (Martin-Smith et al., 2020). These outcome variations may be attributed to differences in intervention protocols and participant characteristics. Including highly intense, low-volume sessions in our analysis might have contributed disproportionately larger improvements among less-fit obese adolescents. Additionally, the relatively small number of included studies might have inflated effect sizes, introducing uncertainty into these findings.

In discussions concerning CRF enhancement, HIIT and MICT have consistently been central themes (Milanović et al., 2015). It is noteworthy that this prior meta-analysis predominantly included higher-volume HIIT protocols (typically >20 min per session) and healthy adult populations, whereas our study specifically isolated the effects of time-efficient LV-HIIT in adolescents with obesity. Our findings further substantiate the effectiveness of low-volume, time-efficient HIIT in enhancing CRF. Specifically, our results indicate that LV-HIIT may be superior to traditional aerobic training methods for improving CRF. Russomando et al. also reported significant increases in VO_2_max in the HIIT group compared with MICT (Russomando et al., 2020). The relatively greater magnitude of improvements observed in our meta-analysis suggests that obese adolescents might respond more sensitively to intense interval stimuli; notably, both higher-volume, high-intensity HIIT and lower-volume, shorter-duration HIIT protocols can yield comparable cardiorespiratory enhancements. This finding holds practical significance: brief HIIT protocols can meaningfully enhance the health-related outcomes of school-based physical education, effectively improving CRF within limited class durations (Liu et al., 2020). Notably, when data from adult studies are pooled, specific investigations have reported no significant differences between HIIT and MICT regarding improvements in VO_2_max (Khodadadi et al., 2023). Therefore, our findings confirm that LV-HIIT protocols tailored explicitly for obese adolescents may enhance cardiorespiratory health more effectively than traditional endurance training methods. This potential advantage may depend on population-specific characteristics and specific training protocols; thus, further targeted research is warranted to confirm these observations.

Our comparison with a non-exercise control group demonstrated that a period of LV-HIIT significantly reduced resting blood pressure (BP) among obese adolescents. SBP decreased markedly relative to controls, and DBP fell in parallel. This finding aligns with earlier evidence; a meta-analysis of overweight adolescents reported significant reductions in SBP (SMD = −0.71, 95% CI [−1.33,−0.09], *p* = 0.02) and DBP (SMD = −0.88, 95% CI [−1.50, −0.25], *p* < 0.01) compared with no-exercise controls (Tozo et al., 2025). Similar antihypertensive effects have been observed in hypertensive adolescents, where HIIT induced a mean SBP reduction of approximately 12.8 mmHg—substantially greater than that observed in control subjects (Popowczak et al., 2022). This finding is further supported by recent evidence in obese adolescents, where short-term HIIT significantly improved cardiovascular profiles, including reductions in systolic blood pressure and optimization of lipid metabolism (Racil et al., 2023). Notably, the BP-lowering effect of brief high-intensity bouts appears larger in individuals with elevated baseline BP, potentially due to greater vascular tone stimulation followed by a pronounced post-exercise hypotensive response (Li et al., 2022). These mechanisms may account for the marked BP reductions observed with low-volume HIIT in obese youths; high-intensity intervals enhance endothelial function and reduce peripheral resistance, conferring benefits comparable to those of longer, conventional exercise sessions (K et al., 2024).

When LV-HIIT was compared with MICT, both interventions effectively reduced BP in obese adolescents, producing comparable moderate decreases in SBP and DBP. In controlled total exercise volume, LV-HIIT did not compromise the antihypertensive effect. The magnitude of BP reduction was similar to that achieved with MICT. A meta-analysis of RCTs involving 309 obese children found that SBP decreased by about 4 mmHg in the HIIT group and 3 mmHg in the MICT group relative to baseline, with no significant difference between groups. Likewise, both groups exhibited a mean DBP reduction of around three mmHg, with similar degrees of improvement (Liu et al., 2020). Variability among study findings may reflect differences in intervention protocols and participant characteristics, as training intensity, interval structure, and recovery duration can modulate outcomes. Indeed, physiological responses to interval training are strongly dependent on the precise manipulation of work-to-rest ratios and recovery intervals (Padulo et al., 2015). Consistent with this, experimental work has shown that differences in interval configuration and intensity prescription can significantly influence adaptations such as VO_2_max and SBP (Cao et al., 2021). Baseline BP status also acts as a key effect modifier. Pre-hypertensive individuals typically demonstrate greater reductions in blood pressure following high-intensity stimuli, whereas normotensive individuals often exhibit responses comparable to those elicited by MICT (Clark et al., 2020). Thus, even at lower exercise volumes, high-intensity intervals remain effective for BP improvement in obese adolescents, underscoring the pivotal role of structured physical activity in reducing cardiovascular risk.

### 4.2 LV-HIIT produces noteworthy improvements in adolescent body composition

We observed significant reductions in BMI, body fat percentage, and waist circumference in adolescents following the LV-HIIT intervention. HIIT induces pronounced excess post-exercise oxygen consumption (EPOC) and elevates catecholamine secretion, collectively increasing energy expenditure and fat oxidation, thereby promoting lipolysis and fat reduction (Jiang et al., 2024; Kolnes et al., 2021). These physiological responses are dose-dependent. Insufficient total training load may result in negligible fat-loss effects. Indeed, studies employing LV-HIIT protocols of ≤500 MET-min·week^−1^ in obese adults found no significant differences in fat reduction compared with control groups (Boer and Moss, 2016; Higgins et al., 2015; Macpherson et al., 2011). These findings suggest that achieving meaningful fat-loss benefits requires HIIT to meet specific training intensity and frequency thresholds. Our study defined LV-HIIT as sessions involving less than 15 min of active high-intensity intervals, effectively maintaining an appropriate intensity–dose balance.

LV-HIIT elicited improvements in BMI, fat mass, body fat percentage, and waist circumference that were comparable to those of MICT. Despite a lower total workload, LV-HIIT achieved similar body-composition improvements as MICT, requiring only one-third to one-half of the weekly time commitment. Comparable results have been reported in adult populations, with HIIT and continuous aerobic training leading to significant weight loss, although differences between methods are minimal. Wewege et al. reported that both HIIT and MICT reduced total fat mass by approximately 1–2 kg and decreased waist circumference among overweight or obese adults, with neither approach demonstrating superior efficacy (Wewege et al., 2017). Similarly, studies involving obese adolescents report no significant differences between HIIT and MICT regarding improvements in body fat percentage, BMI, or related anthropometric indices (Liu et al., 2020; Cao et al., 2022b). Such time-efficient yet effective training modalities are especially attractive to students who must manage demanding academic schedules (Yin H. et al., 2025). Some researchers have mentioned that if HIIT volume is excessively low, fat-loss effects may be less pronounced than energy-matched MICT. This suggests that insufficient energy expenditure can limit the cumulative fat-burning efficacy of brief, high-intensity exercise sessions (Dias et al., 2018). However, our meta-regression analysis did not find a significant dose-response relationship between training volume parameters and fat loss (*p* > 0.05 for all; Supplementary Figure S1), although the small number of studies limits the statistical power to detect such effects. With matched total exercise volumes, HIIT may have a more favorable effect on BMI in young adults than MICT. Nevertheless, outcomes are often influenced by training frequency, session duration, and participant demographics (Song et al., 2024; Zhu et al., 2021). Given that insufficient time is frequently cited as a primary barrier to adopting active lifestyles among obese individuals (Andersen and Jakicic, 2009), our key finding that even reduced-volume HIIT protocols can significantly improve body composition in obese adolescents has important practical implications. The specific exercise modality employed also critically influences outcomes, as weight-bearing aerobic activities like running generally involve greater muscle recruitment and energy expenditure than non-weight-bearing activities such as cycling. Some studies have shown that running-based HIIT protocols reduce body fat more effectively than cycling-based protocols (Khodadadi et al., 2023). Although fat loss between HIIT and MICT may be comparable, HIIT generally confers additional metabolic benefits. Indeed, our LV-HIIT protocol yielded additional improvements in CRF and cardiovascular health indicators beyond those attained with MICT.

Our meta-regression analysis further revealed a marginal moderating effect of baseline obesity status on BMI outcomes. Participants with a lower baseline BMI (<30 kg/m^2^) achieved a greater reduction in BMI from the LV-HIIT intervention, while those with severe obesity showed a smaller decrease. This trend did not reach statistical significance, indicating that a lower baseline BMI may be associated with a greater BMI reduction following LV-HIIT intervention. From a practical perspective, our findings suggest that LV-HIIT may be particularly beneficial for children and adolescents with relatively mild obesity. This specificity aligns with the call for personalized exercise prescriptions based on initial obesity levels. The boundary effect observed in this regression analysis is not isolated; it is consistent with findings from the subgroup analysis of this study regarding baseline BMI. This finding is consistent with high-quality research on adolescent weight management through exercise interventions, especially HIIT. Costigan et al. pointed out that HIIT can improve health-related fitness in overweight and obese adolescents, and baseline characteristics may influence outcomes (Costigan et al., 2015). Poon et al. emphasized that HIIT has universal benefits in improving cardiopulmonary health in children and adolescents, but individual baseline conditions may have a greater influence (Poon et al., 2024). Therefore, promoting LV-HIIT programs among moderately overweight adolescents may lead to significant improvements in BMI and represent a promising, resource-efficient weight management strategy for this subgroup.

The borderline trend (*p* = 0.05) suggesting that lower baseline BMI may predict greater BMI reductions should be interpreted with caution. Our analysis was likely underpowered given the limited number of trials, and unmeasured confounders (e.g., diet, pubertal status) may partly explain this pattern. Notably, previous studies have reported opposite findings, associating higher baseline BMI with greater reduction (Imperiali et al., 2025; Su et al., 2025). Given these inconsistencies, this finding should be considered hypothesis-generating rather than conclusive, and confirmation in adequately powered trials or individual participant data meta-analyses is required.

### 4.3 Limitations

This study has several limitations that warrant further investigation. First, this meta-analysis incorporated only 12 peer-reviewed studies; this relatively small sample may limit the robustness and generalizability of our conclusions. Second, considerable heterogeneity was observed among studies for specific outcomes. Although random-effects models, sensitivity analyses, and subgroup analyses were systematically conducted to identify sources of heterogeneity, underlying confounding variables—such as differences in regional or cultural contexts and experimental designs—may not have been entirely accounted for. Third, measurement reliability for primary outcomes was inadequately reported in the included trials, which may have introduced additional measurement error and potentially attenuated the true intervention effects. Fourth, meta-regression analyses with a limited number of studies have low statistical power to detect true moderator effects and are prone to false negatives. The borderline finding for baseline BMI (*p* = 0.05) should be interpreted with particular caution given only 10 studies contributed data. While this meets the minimum of 10 studies per covariate typically recommended for meta-regression, the analysis remains susceptible to limited statistical power. Fifth, standard deviations for change scores were imputed assuming a correlation coefficient of 0.5; although sensitivity analyses confirmed the robustness of our results, actual correlations were not available. Moreover, the lack of individual participant data (IPD) limited our ability to explore participant-level moderators. Specifically, although BMI Z-score is the preferred metric for pediatric obesity, most included studies only reported raw BMI values. Consequently, we were unable to conduct analyses based on Z-scores, and the use of raw BMI (with a cut-off of 30 kg/m^2^ for subgrouping) may not fully capture age- and sex-specific adiposity status, particularly in diverse ethnic populations. Additionally, funnel plot asymmetry detected for BMI and VO_2_max outcomes suggests possible publication bias favoring positive findings, meaning the true effects may be smaller than estimated. However, alternative explanations including small-study effects and genuine heterogeneity cannot be excluded. Furthermore, we acknowledge that certain other obesity-related indicators, such as metabolic markers (e.g., blood lipids, glycemic traits), were not included in this meta-analysis; future research should aim to evaluate these diverse profiles as more standardized trials become available. Lastly, most studies included in this meta-analysis employed short-term interventions (≤12 weeks), limiting our capacity to evaluate the long-term effectiveness of LV-HIIT on cardiovascular disease risk and trajectories of weight regain.

## 5 Conclusion

Overall, our synthesis indicates that LV-HIIT effectively improves body composition and cardiometabolic health, yielding significant enhancements in CRF. Compared to MICT, LV-HIIT delivers comparable health benefits while significantly reducing total training time by 24%–50%, making it an efficient and viable alternative exercise modality.

## Data Availability

The original contributions presented in the study are included in the article/Supplementary Material, further inquiries can be directed to the corresponding author.
