# Supplementary material for: Time-efficient and beneficial strategy: low-volume high-intensity interval training for cardiometabolic health and body composition outcomes in children and adolescents with overweight or obesity—a systematic review and meta-analysis
Source: Front Physiol. 2026 Jan 30;16:1732253. doi: 10.3389/fphys.2025.1732253 (PMC12900728; doi:10.3389/fphys.2025.1732253)
Supplement: Supplementary file 1 [file Table1.docx]

Supplementary Material

Supplementary Material PRISMA checklist

| **Section and Topic** | **Item #** | **Checklist item** | **Location where item is reported** |
| --- | --- | --- | --- |
| **Time-efficient and beneficial strategy: low-volume high-intensity interval training for cardiometabolic health and body composition outcomes in children and adolescents with overweight or obesity—a systematic review and meta-analysis** | | |  |
| Title | 1 | Identify the report as a systematic review, meta-analysis, or both. | Title |
|  | | |  |
| Structured summary | 2 | Provide a structured summary including, as applicable: background; objectives; data sources; study eligibility criteria, participants, and interventions; study appraisal and synthesis methods; results; limitations; conclusions and implications of key findings; systematic review registration number. | Abstract |
| **INTRODUCTION** | | |  |
| Rationale | 3 | Describe the rationale for the review in the context of what is already known. | 1.Introduction  1.Introduction |
| Objectives | 4 | Provide an explicit statement of questions being addressed with reference to participants, interventions, comparisons, outcomes, and study design （PICOS） . |  |
| **METHODS** | | |  |
| Protocol and registration | 5 | Indicate if a review protocol exists, if and where it can be accessed （e.g., Web address）, and, if available, provide registration information, including registration number. | 2.1 |
| Eligibility criteria | 6 | Specify study characteristics （e.g., PICOS, length of follow-up） and report characteristics （e.g., years considered,language, publication status）used as criteria for eligibility, giving rationale. | 2.2-2.3 |
| Information sources | 7 | Describe all information sources （e.g., databases with dates of coverage, contact with study authors to identify additional studies） in the search and date last searched. | 2.2 |

| Search | 8 | Present a full electronic search strategy for at least one database, including any limits used, such that it could be repeated. | 2.2 |
| --- | --- | --- | --- |
| Study selection | 9 | State the process for selecting studies （i.e., screening, eligibility, included in systematic review, and, if applicable,included in the meta-analysis）. | 2.3-2.4 |
| Data collection process | 10 | Describe the method of data extraction from reports （e.g., piloted forms, independently, in duplicate） and any processes for obtaining and confirming data from investigators. | 2.4 |
| Data items | 11 | List and define all variables for which data were sought （e.g., PICOS, funding sources） and any assumptions and simplifications made. | 2.3-2.5 |
| Risk of bias in individual studies | 12 | Describe methods used for assessing risk of bias of individual studies （including specification of whether this was done at the study or outcome level）, and how this information is to be used in any data synthesis. | 2.6 |
| Summary measures | 13 | State the principal summary measures (e.g., risk ratio, difference in means). | 2.7 |
| Synthesis of results | 14 | Describe the methods of handling data and combining results of studies, if done, including measures of consistency （e.g., I2） for each meta-analysis. | 2.7 |

| Risk of bias across studies | 15 | Specify any assessment of risk of bias that may affect the cumulative evidence （e.g., publication bias, selective reporting within studies）. | 2.7-2.8 |
| --- | --- | --- | --- |
| Additional analyses | 16 | Describe methods of additional analyses （e.g., sensitivity or subgroup analyses, meta-regression）, if done, indicating which were pre-specified. | 2.7 |
| **RESULTS** | | |  |
| Study selection | 17 | Give the numbers of studies screened, assessed for eligibility, and included in the review, with reasons for exclusions at each stage, ideally with a flow diagram. | 3.1 |

| Study characteristics | 18 | For each study, present characteristics for which data were extracted（e.g., study size, PICOS, follow-up period） and provide the citations. | 3.2-3.3 |
| --- | --- | --- | --- |
| Risk of bias within studies | 19 | Present data on the risk of bias of each study and, if available, any outcome level assessment （see item 12）. | 3.6 |
| Results of individual studies | 20 | For all outcomes considered （benefits or harms）, present, for each study: （a） simple summary data for each intervention group, （b） effect estimates and confidence intervals, ideally with a forest plot. | 3.4-3.5 |
| Synthesis of results | 21 | Present the results of each meta-analysis done, including confidence intervals and measures of consistency. | 3.4-3.5 |
| Risk of bias across studies | 22 | Present results of any assessment of risk of bias across studies （see Item 15）. | 3.6 |
| Additional analysis | 23 | Give results of additional analyses, if done （e.g., sensitivity or subgroup analyses, meta-regression [see Item 16]）. | 3.4-3.7 |
| **DISCUSSION** | | |  |
| Summary of evidence | 24 | Summarize the main findings, including the strength of evidence for each main outcome; consider their relevance to key groups （e.g., healthcare providers, users, and policymakers）. | 4.Discussion |
| Limitations | 25 | Discuss limitations at study and outcome level （e.g., risk of bias）, and at review-level （e.g., incomplete retrieval of identified research, reporting bias）. | 4.3 |
| Conclusions | 26 | Provide a general interpretation of the results in the context of other evidence, and implications for future research. | 5.Conclusions |
| **FUNDING** | | |  |
| Funding | 27 | Describe sources of funding for the systematic review and other support （e.g., supply of data）; role funders for the systematic review. | Funding |

**Supplementary Table 2 Search Strategy**

| **Database** | **Search strategy** | **Result** |
| --- | --- | --- |
| Web of Science | 1= ((((((((((TS=(Adolescent)) OR TS=(child)) OR TS=(Adolescents)) OR TS=(Adolescence)) OR TS=(Youth)) OR TS=(Youths)) OR TS=(Teens)) OR TS=(Teen)) OR TS=(Teenagers)) OR TS=(Teenager)) OR TS=(Children) and Preprint Citation Index (Exclude – Database)  2= (((TS=(randomized controlled trial)) OR TS=(randomized)) OR TS=(placebo)) OR TS=(RCT) and Preprint Citation Index (Exclude – Database)  3=((((((((((((TS=(body composition)) OR TS=(body component )) OR TS=(lean body mass)) OR TS=(body weight)) OR TS=(body adiposity index)) OR TS=(body fat)) OR TS=(body fat percentage)) OR TS=(blood pressure)) OR TS=(VO2max)) OR TS=(fitness)) OR TS=(CRF)) OR TS=(VO2peak)) OR TS=(MetS z-score) and Preprint Citation Index (Exclude – Database)  4=((((((((((((TS=(high-intensity interval training)) OR TS=(high intensity interval training)) OR TS=(high-intensity interval training)) OR TS=(high intensity interval exercise)) OR TS=(high-intensity interval exercise)) OR TS=(high-intensity intermittent exercise)) OR TS=(high intensity intermittent exercise)) OR TS=(SIT)) OR TS=(Low-volume HIIT)) OR TS=(Low-volume high-intensity interval training)) OR TS=(HIIT)) OR TS=(HIIE)) OR TS=(Sprint Interval Training) and Preprint Citation Index (Exclude – Database)  4 AND 3 AND 2 AND 1 | 790 |
| PubMed | ((((("Exercise"[Mesh]) OR (((((((((((((((((((((((Physical Activity[Title/Abstract]) OR (Activities, Physical[Title/Abstract])) OR (Activity, Physical[Title/Abstract])) OR (Physical Activities[Title/Abstract])) OR (Exercise*, Physical[Title/Abstract])) OR (Physical Exercise*[Title/Abstract])) OR (Aerobic Exercise*[Title/Abstract])) OR (Exercise* Training[Title/Abstract])) OR (Training, Exercise*[Title/Abstract])) OR (resistance training[Title/Abstract])) OR (strength training[Title/Abstract])) OR (combined training[Title/Abstract])) OR (sprint interval training[Title/Abstract])) OR (high intensity interval training[Title/Abstract])) OR (Acute Exercise*[Title/Abstract])) OR (Exercise*, Acute[Title/Abstract])) OR (Isometric Exercise*[Title/Abstract])) OR (Exercise, Isometric[Title/Abstract])) OR (endurance training[Title/Abstract])) OR (concurrent training[Title/Abstract])) OR (interval training[Title/Abstract])) OR (circuit training[Title/Abstract])))) AND (("Body Composition"[Mesh]) OR ((((((((((((((body component[Title/Abstract]) OR (lean body mass[Title/Abstract])) OR (body weight[Title/Abstract])) OR (body adiposity index[Title/Abstract])) OR (body fat[Title/Abstract])) OR (body fat percentage[Title/Abstract])) OR (Body Compositions[Title/Abstract])) OR (Composition, Body[Title/Abstract])) OR (body weight[Title/Abstract])) OR (body mass index[Title/Abstract])) OR (body fat percentage[Title/Abstract])) OR (body fat[Title/Abstract])) OR (fat mass[Title/Abstract])) OR (body mass[Title/Abstract])))) AND (((("Obesity"[Mesh]) OR ((overweight[Title/Abstract]) OR (obese[Title/Abstract]))) OR ("Pediatric Obesity"[Mesh])) OR (((((((((((((((Obesity, Pediatric[Title/Abstract]) OR (Obesity in Childhood[Title/Abstract])) OR (Childhood Onset Obesity[Title/Abstract])) OR (Obesity, Childhood Onset[Title/Abstract])) OR (Child Obesity[Title/Abstract])) OR (Obesity, Child[Title/Abstract])) OR (Childhood Obesity[Title/Abstract])) OR (Obesity, Childhood[Title/Abstract])) OR (Adolescent Obesity[Title/Abstract])) OR (Obesity, Adolescent[Title/Abstract])) OR (Obesity in Adolescence[Title/Abstract])) OR (Childhood Overweight[Title/Abstract])) OR (Overweight, Childhood[Title/Abstract])) OR (Adolescent Overweight[Title/Abstract])) OR (Overweight, Adolescent[Title/Abstract])))) AND (("Adolescent"[Mesh]) OR (((((Adolescents[Title/Abstract]) OR (Adolescence[Title/Abstract])) OR (Youth*[Title/Abstract])) OR (Teen*[Title/Abstract])) OR (Teenager*[Title/Abstract])))) AND (((randomized controlled trial[Title/Abstract]) OR (randomized[Title/Abstract])) OR (placebo[Title/Abstract])) | 209 |
| EBSCO | AB Adolescent OR AB Adolescents OR AB Adolescence OR AB Youth OR AB Youths OR AB Teens OR AB Teen OR AB Teenagers OR AB Teenager OR AB Children OR AB Child OR  AB high intensity interval training OR AB high-intensity interval training OR AB high intensity interval exercise OR AB high-intensity interval exercise OR AB high-intensity intermittent exercise OR AB high intensity intermittent exercise OR AB SIT OR AB Low-volume HIIT OR AB Low-volume high-intensity interval training OR AB HIIT OR AB HIIE OR  AB body component OR AB lean body mass OR AB body weight OR AB body adiposity index OR AB body fat OR AB body fat percentage OR AB body composition OR AB body composition OR AB blood pressure OR AB fitness OR AB VO2max OR AB CRF OR AB VO2peak OR AB MetS z-score OR  AB randomized controlled trial OR AB randomized OR AB placebo OR | 115 |
| Embase | 1='Obesity, Pediatric':ab,ti or 'Obesity in Childhood':ab,ti or 'Childhood Onset Obesity':ab,ti or 'Obesity, Childhood Onset':ab,ti or 'Obesity, Child':ab,ti or 'Pediatric Obesity':ab,ti or 'Childhood Obesity':ab,ti or 'Obesity, Childhood':ab,ti or 'Adolescent Obesity':ab,ti or 'Obesity, Adolescent':ab,ti or 'Obesity in Adolescence':ab,ti or 'Childhood Overweight':ab,ti or 'Overweight, Childhood':ab,ti or 'Adolescent Overweight':ab,ti or  2='high intensity interval training':ab,ti or 'high-intensity interval training':ab,ti or 'high intensity interval exercise':ab,ti or 'high-intensity interval exercise':ab,ti or 'high-intensity intermittent exercise':ab,ti or 'high intensity intermittent exercise':ab,ti or 'SIT':ab,ti or 'Low-volume HIIT':ab,ti or 'Low-volume high-intensity interval training':ab,ti or 'HIIT':ab,ti or 'HIIE':ab,ti or  3='body component ':ab,ti or 'lean body mass':ab,ti or 'body weight':ab,ti or 'body adiposity index':ab,ti or 'body fat':ab,ti or 'body fat percentage':ab,ti or 'blood pressure':ab,ti or 'VO2max':ab,ti or 'fitness':ab,ti or 'CRF':ab,ti or 'VO2peak':ab,ti or 'MetS z-score':ab,ti or  4='randomized controlled trial':ab,ti or 'randomized':ab,ti or 'placebo':ab,ti or  5='Adolescents':ab,ti or 'Adolescence':ab,ti or 'Youth':ab,ti or 'Youths':ab,ti or 'Teens':ab,ti or 'Teen':ab,ti or 'Teenagers':ab,ti or 'Teenager':ab,ti or 'Children':ab,ti or  1 AND 2 AND 3 AND 4 AND 5 | 186 |
| Cochrane | 1=(Obesity, Pediatric):ti,ab,kw or (Obesity in Childhood):ti,ab,kw or (Childhood Onset Obesity):ti,ab,kw or (Obesity, Childhood Onset):ti,ab,kw or (Child Obesity):ti,ab,kw or (Obesity, Child):ti,ab,kw or (Childhood Obesity):ti,ab,kw or (Obesity, Childhood):ti,ab,kw or (Adolescent Obesity):ti,ab,kw or (Obesity, Adolescent):ti,ab,kw or (Obesity in Adolescence):ti,ab,kw or (Overweight, Childhood):ti,ab,kw or (Childhood Overweight):ti,ab,kw or (Adolescent Overweight):ti,ab,kw or  2=(high intensity interval training):ti,ab,kw or (high-intensity interval training):ti,ab,kw or (high intensity interval exercise):ti,ab,kw or (high-intensity interval exercise):ti,ab,kw or (high intensity intermittent exercise):ti,ab,kw or (high-intensity intermittent exercise):ti,ab,kw or (SIT):ti,ab,kw or (Low-volume HIIT):ti,ab,kw or (Low-volume high-intensity interval training):ti,ab,kw or (HIIT):ti,ab,kw or (HIIE):ti,ab,kw or  3=(body component ):ti,ab,kw or (lean body mass):ti,ab,kw or (body weight):ti,ab,kw or (body adiposity index):ti,ab,kw or (body fat):ti,ab,kw or (body fat percentage):ti,ab,kw or (body mass):ti,ab,kw or (BMI):ti,ab,kw or (waist circumference):ti,ab,kw or (hip circumference):ti,ab,kw or (waist-to-hip ratio):ti,ab,kw or (% body fat):ti,ab,kw or (blood pressure):ti,ab,kw or (VO2max):ti,ab,kw or  4=(Adolescents):ti,ab,kw or (Adolescence):ti,ab,kw or (Youth):ti,ab,kw or (Youths):ti,ab,kw or (Teen):ti,ab,kw or (Teens):ti,ab,kw or (Teenagers):ti,ab,kw or (Teenager):ti,ab,kw or (Children):ti,ab,kw or  1 AND 2 AND 3 AND 4 | 697 |
| CNKI | 篇关摘:超重+肥胖(精确))AND(篇关摘:儿童+青少年+未成年人(精确))AND(篇关摘:高强度间歇运动+HIT+低容量高强度间歇运动(精确))AND(篇关摘:体成分+身体成分+体重+BMI+瘦体重(精确))AND(篇关摘:随机对照实验+随机+RCT(精确)) | 188 |

**Supplementary Table 3.** Sensitivity analyses of the effects of LV-HIIT on health outcomes using different correlation coefficients (*r*) for imputing change-score standard deviations.

| **Outcomes** | **Comparison** | **r=0.50 (Main Analysis)** | **r=0.30 (Conservative)** | **r=0.70 (Liberal)** | **Robustness Check** |
| --- | --- | --- | --- | --- | --- |
|  | **Groups** | **SMD (95% CI); I²** | **SMD (95% CI); I²** | **SMD (95% CI); I²** | **Result Changed** |
| **BMI** | LV-HIIT vs CON | -1.15 (-1.68, -0.61); 77% | -0.99 (-1.46, -0.52); 72% | -1.43 (-2.06, -0.79); 82% | No |
|  | LV-HIIT vs MICT | -0.27 (-0.49, -0.04); 0% | -0.23 (-0.46, -0.01); 0% | -0.34 (-0.56, -0.11); 0% | No |
| **Body fat percentage** | LV-HIIT vs CON | -0.84 (-1.09, -0.59); 0% | -0.72 (-0.96, -0.47); 0% | -1.08 (-1.33, -0.82); 0% | No |
|  | LV-HIIT vs MICT | -0.06 (-0.35, 0.22); 20% | -0.05 (-0.31, 0.20); 0% | -0.08 (-0.45, 0.28); 49% | No |
| **Waist circumference** | LV-HIIT vs CON | -0.62 (-0.93, -0.32); 0% | -0.53 (-0.83, -0.23); 0% | -0.80 (-1.17, -0.43); 27% | No |
|  | LV-HIIT vs MICT | -0.37 (-0.75, 0.01); 0% | -0.32 (-0.70, 0.06); 0% | -0.47 (-0.85, -0.09); 0% | Yes |
| **SBP** | LV-HIIT vs CON | -0.80 (-1.11, -0.49); 0% | -0.68 (-0.99, -0.38); 0% | -1.01 (-1.33, -0.69); 0% | No |
|  | LV-HIIT vs MICT | -0.25 (-0.55, -0.05); 0% | -0.21 (-0.51, 0.08); 0% | -0.31 (-0.61, -0.01); 0% | Yes |
| **DBP** | LV-HIIT vs CON | -0.47 (-0.77, -0.17); 0% | -0.41 (-0.71, -0.10); 0% | -0.58 (-0.89, -0.28); 0% | No |
|  | LV-HIIT vs MICT | 0.07 (-0.23, 0.36); 0% | 0.06 (-0.24, 0.35); 0% | 0.09 (-0.21, 0.38); 0% | No |
| **VO_2_max** | LV-HIIT vs CON | 2.10 (1.32, 2.87); 73% | 1.79 (1.13, 2.46); 67% | 2.59 (1.65, 3.53); 78% | No |
|  | LV-HIIT vs MICT | 0.76 (0.39, 1.13); 37% | 0.64 (0.32, 0.95); 17% | 0.97 (0.51, 1.43); 56% | No |

Abbreviations: *CI*, confidence interval; *CON*, non-exercising control; *SBP*, Systolic blood pressure; *DBP*, diastolic blood pressure; *LV-HIIT*, low-volume high-intensity interval training; *MICT*, moderate-intensity continuous training; *SMD*, standardized mean difference.

Notes: Values are pooled standardized mean differences (SMDs) with 95% confidence intervals (CIs) and *I²* statistics from random-effects meta-analyses based on change scores. For trials that did not report change-score standard deviations, the *SD* of change was imputed from baseline and post-intervention *SDs* using an assumed pre–post correlation coefficient *r*. The primary analyses used *r* = 0.50, whereas *r* = 0.30 (more conservative, yielding larger *SD_change*) and r = 0.70 (more liberal, yielding smaller *SD_change*) were applied in the sensitivity analyses. The “Robustness check” column indicates whether the statistical significance (*p* < 0.05) and direction of the pooled effect remained consistent across all *r* values (“No”) or were sensitive to the choice of *r* (“Yes”). Negative SMDs indicate greater reductions in the outcome favouring LV-HIIT over the comparator.

**Supplementary Table 4 Outcome measurement protocols and equipment specifications of included studies**

| **Study** | **Outcome** | **Measurement Method / Protocol** | **Device Specifications (Model, Manufacturer,Country)** |
| --- | --- | --- | --- |
| **Abassi et al. 2025** | BMI | Calculated from measured mass and height | **Mass:** Diagnostic Scale (BC-533, Tanita, Japan) |
|  |  |  | **Height:** Stadiometer |
|  | Body fat percentage | BIA | Diagnostic Scale (BC-533, Tanita, Japan) |
|  | Waist circumference | Measured between the lower rib margin and iliac crest | Non-deformable tape ruler |
|  | Blood pressure | Seated position after 10 min rest | Electronic Monitor (BP652, Omron, USA) |
| **Su et al. 2024** | BMI | Calculated from measured mass and height | **Mass:** Electronic scale |
|  |  |  | **Height:** Stadiometer (Model 213, Seca, Germany) |
|  | Blood pressure | Seated position after ≥ 5 min rest; 3 measurements | Manual Sphygmomanometer (Accoson, UK) |
|  | VO_2_max | Direct: Treadmill GXT | Metabolic System (VO2000, Medical Graphics, USA) |
| **Cao et al. 2024** | BMI | Calculated from measured mass and height | **Mass:** Body Composition Analyzer (InBody 770, Biospace, Korea) |
|  |  |  | **Height:** Stadiometer |
|  | Body fat percentage | DXA | DXA Scanner (Lunar Prodigy, GE Healthcare, USA) |
|  | VO_2_max | Direct: Treadmill GXT | Portable Gas Analyzer (K5, Cosmed, Italy) |
| **Zuo et al. 2023** | BMI | Calculated from measured mass and height | **Mass:** Electronic scale |
|  |  |  | **Height:** Stadiometer |
|  | Body fat percentage | BIA | Body Composition Analyzer (JAWON, GAIA KIKO, Korea) |
|  | Blood pressure | Seated position after 5 min rest; mean of ≥ 2 measurements | Electronic Monitor (BP652, Omron, USA) |
|  | VO_2_max | Direct: Treadmill GXT | Metabolic System (Metalyzer 3B, Cortex, Germany) |
| **Abassi et al. 2023** | BMI | Calculated from measured mass and height | **Mass:** Diagnostic Scale (BG39, Beurer, Japan) |
|  |  |  | **Height:** Stadiometer (Model 214, Seca, Germany) |
|  | Body fat percentage | BIA | Diagnostic Scale (BG39, Beurer, Japan) |
|  | Waist circumference | Measured between the lower rib margin and iliac crest | Non-deformable tape ruler |
| **Li et al. 2023** | BMI | Calculated from measured mass and height | **Mass:** Diagnostic Scale (BC-533, Tanita, Japan) |
|  |  |  | **Height:** Stadiometer |
|  | Body fat percentage | DXA | DXA Scanner (Lunar Prodigy, GE Healthcare, USA) |
|  | Waist circumference | Measured between the lower rib margin and iliac crest | Non-deformable tape ruler |
|  | Blood pressure | Seated position after 10 min rest; lowest of 3 measurements | Electronic Monitor (HBP-1300, Omron, Japan) |
|  | VO_2_max | Indirect: 20-m Shuttle Run Test | Field-based prediction equations |
| **Abassi et al. 2022** | BMI | Calculated from measured mass and height | **Mass:** Diagnostic Scale (BG39, Beurer, Japan) |
|  |  |  | **Height:** Stadiometer (Model 214, Seca, Germany) |
|  | Body fat percentage | BIA | Diagnostic Scale (BG39, Beurer, Japan) |
|  | Waist circumference | Measured between the lower rib margin and iliac crest | Non-deformable tape ruler |
|  | Blood pressure | Seated position after 15 min rest | Arm Tensiometer (Exacto KD 591, Biosynex, France) |
| **Cao et al. 2022a** | BMI | Calculated from measured mass and height | **Mass:** Body Composition Analyzer (InBody 770, Biospace, Korea) |
|  |  |  | **Height:** Stadiometer |
|  | Body fat percentage | DXA | DXA Scanner (Lunar Prodigy, GE Healthcare, USA) |
|  | Waist circumference | Measured between the lower rib margin and iliac crest | Non-deformable tape ruler |
|  | Blood pressure | Seated position after ＞5 min rest | Pulse Wave BP Monitor (RBP-9000, Raycome, China) |
|  | VO_2_max | Indirect: 20-m Shuttle Run Test | Field-based prediction equations |
| **Cao et al. 2022b** | BMI | Calculated from measured mass and height | **Mass:** Diagnostic Scale (BC-533, Tanita, Japan) |
|  |  |  | **Height:** Stadiometer (RGZ-120-RT, Shanghai, China) |
|  | Body fat percentage | DXA | DXA Scanner (Lunar Prodigy, GE Healthcare, USA) |
|  | Waist circumference | Measured between the lower rib margin and iliac crest | Non-deformable tape ruler |
|  | Blood pressure | Seated position after ＞5 min rest | Electronic Monitor (BP652, Omron, USA) |
|  | VO_2_max | Direct: Treadmill GXT | Metabolic System (MAX-IIa, AEI Technologies, USA) |
| **Cao et al. 2022c** | BMI | Calculated from measured mass and height | **Mass:** Body Composition Analyzer (InBody 770, Biospace, Korea) |
|  |  |  | **Height:** Stadiometer |
|  | Body fat percentage | BIA | Body Composition Analyzer (InBody 770, Biospace, Korea) |
|  | VO_2_max | Indirect: 20-m Shuttle Run Test | Field-based prediction equations |
| **Paahoo et al. 2020** | BMI | Calculated from measured mass and height | **Mass:** Diagnostic Scale (BC-533, Tanita, Japan) |
|  |  |  | **Height:** Stadiometer (Model 217, Seca, Germany) |
|  | Body fat percentage | Skinfold thickness (3 sites: triceps, subscapular, leg) | Skinfold Caliper (Harpenden, UK) |
|  | Waist circumference | Measured between the lower rib margin and iliac crest | Non-elastic tapemeter |
| **Cvetkovićet al. 2018** | BMI | Calculated from measured mass and height | **Mass:** Body Composition Analyzer (InBody 720, Biospace, Korea) |
|  |  |  | **Height:** Stadiometer (Model 220, Seca, Germany) |
|  | Body fat percentage | BIA | Body Composition Analyzer (InBody 720, Biospace, Korea) |
|  | Blood pressure | Seated position after 5 min rest | Manual Sphygmomanometer (Standard) |

Abbreviations: *BIA*, bioelectrical impedance analysis; *BMI*, body mass index; *BP*, blood pressure; *DXA*, dual-energy X-ray absorptiometry; *GXT*, graded exercise test; *VO₂max*, maximal oxygen uptake.

Notes:

1.Direct assessment of VO₂max refers to laboratory testing utilizing breath-by-breath gas analysis systems.

2.Indirect assessment of VO₂max refers to estimation via field tests using validated prediction equations.

3.Device specifications are presented as: Device Name (Model, Manufacturer, Country). Where specific model numbers or manufacturers were not reported in the original study, the general device type (e.g., "Electronic scale" or "Stadiometer") is listed.

**Supplementary Table 5: GRADE criteria for certainty of evidence on (1) the effectiveness of LV-HIIT vs. CON on health outcomes, (2) the effectiveness of LV-HIIT vs. MICT**

| Outcome | Sample size | Certainty of evidence assessment | | | | | Hedge's g  with (95% CI) | GRADE* |
| --- | --- | --- | --- | --- | --- | --- | --- | --- |
|  |  | Risk of bias | Inconsistency | Indirectness | Imprecision | Others |  |  |
| LV−HIIT vs. No Training | | | | | | | | |
| BMI | 298  (K=10) | Not serious | Not serious | Not serious | Serious | None | −1.15  (−1.68,0.61) | ⨁⨁◯◯ Low |
| BF% | 273  (K=9) | Not serious | Not Serious | Not Serious | Not Serious | None | −0.84  (−1.09,−0.59) | ⨁⨁⨁◯ Moderate |
| WC | 175  (K=6) | Not serious | Not Serious | Not serious | Not Serious | None | −0.62  (−0.93,−0.32) | ⨁⨁⨁◯  Moderate |
| SBP | 175  (K=6) | Not serious | Not Serious | Not serious | Not Serious | None | −0.80  (−1.11,−0.49) | ⨁⨁⨁◯  Moderate |
| DBP | 175  (K=6) | Not serious | Not Serious | Not serious | Not Serious | None | −0.49  (−0.80,−0.19) | ⨁⨁⨁◯ Moderate |
| VO2max | 160  (K=5) | Not serious | Serious | Not serious | Serious | Low  sample  size | 1.93  (−1.27, 2.59) | ⨁◯◯◯ very  Low |
| LV−HIIT vs. MICT | | | | | | | | |
| BMI | 302  (K=10) | Not serious | Not Serious | Not serious | Serious | None | −0.24  (−0.47,0.01) | ⨁⨁◯◯ Low |
| BF% | 237  (K =8) | Not serious | Not Serious | Not Serious | Serious | None | −0.06  (−0.35, 0.22) | ⨁⨁◯◯ Low |
| WC | 108  (K=4) | Not serious | Not Serious | Not serious | Serious | Low  Sample  size | −0.37  (−0.75, 0.01) | ⨁◯◯◯ very  Low |
| SBP | 174  (K=6) | Not serious | Not Serious | Not serious | Serious | None | −0.25  (−0.55, 0.05) | ⨁⨁◯◯  Low |
| DBP | 174  (K=6) | Not serious | Not Serious | Not serious | Serious | None | 0.07  (−0.23, 0.36) | ⨁⨁◯◯  Low |
| VO2max | 199  (K=6) | Not serious | Not Serious | Not serious | Not Serious | None | 0.63  (0.34, 0.91) | ⨁⨁⨁◯  Moderate |

Note: *K*, the total number of effects included in the pooled effect size; *LV-HIIT,* low-volume high-intensity interval training; *MICT,* moderate-intensity continuous training; *BMI,* body mass index; *VO2max*, Maximal Oxygen Uptake; *DBP*, diastolic blood pressure; *SBP*, systolic blood pressure; *GRADE*, Criteria for Certainty of Evidence.

High, Very confident in the estimated effect; Moderate, Moderately confident in the estimated effect; Low, Limited confidence in the estimated effect; Very low, Very limited confidence in the estimated effect

**Supplementary Fig.1:** **Dose-response effect of LV-HIIT on BMI and body fat percentage(SMD): Meta-regression analysis results on predictors related to the training protocol. The size of the circle is proportional to the accuracy of the effect (s) observed in each study. A negative value indicates that LV-HIIT results in a greater reduction in BMI and body fat percentage than the control group. The dotted line represents the 95% confidence interval of the regression line.**

**1. Meta-regression analysis of BMI**

**2. Meta-regression analysis of body fat percentage**

**Supplementary Fig. 2: The risk of bias for each study**

**Supplementary Fig.3: The risk of publication bias**

**Body Mass Index LV-HIIT vs. CON**

**Body Mass Index LV-HIIT vs. MICT**

**Body fat percentage LV-HIIT vs. CON**

**Body fat percentage LV-HIIT vs. MICT**

**Waist circumference LV-HIIT vs. CON**

**Waist circumference LV-HIIT vs. MICT**

**Systolic blood pressure LV-HIIT vs. CON**

**Systolic blood pressure LV-HIIT vs. MICT**

**Diastolic blood pressure LV-HIIT vs. CON**

**Diastolic blood pressure LV-HIIT vs. MICT**

**VO₂max LV-HIIT vs. CON**

**VO₂max LV-HIIT vs. MICT**
